# Emerging therapeutic strategies in glioblastsoma: drug repurposing, mechanisms of resistance, precision medicine, and technological innovations

**DOI:** 10.1007/s10238-025-01631-0

**Published:** 2025-04-13

**Authors:** Mohamed S. Anwer, Mohammed A. Abdel-Rasol, Wael M. El-Sayed

**Affiliations:** Department of Zoology, Faculty of Science, Ain Shams University, Abbassia, Cairo, 11566 Egypt

**Keywords:** Artificial intelligence, Blood–brain barrier, Precision medicine, Nanotechnology, Next-generation sequencing, Tumor microenvironment

## Abstract

Glioblastoma (GBM) is an aggressive Grade IV brain tumor with a poor prognosis. It results from genetic mutations, epigenetic changes, and factors within the tumor microenvironment (TME). Traditional treatments like surgery, radiotherapy, and chemotherapy provide limited survival benefits due to the tumor's heterogeneity and resistance mechanisms. This review examines novel approaches for treating GBM, focusing on repurposing existing medications such as antipsychotics, antidepressants, and statins for their potential anti-GBM effects. Advances in molecular profiling, including next-generation sequencing, artificial intelligence (AI), and nanotechnology-based drug delivery, are transforming GBM diagnosis and treatment. The TME, particularly GBM stem cells and immune evasion, plays a key role in therapeutic resistance. Integrating multi-omics data and applying precision medicine show promise, especially in combination therapies and immunotherapies, to enhance clinical outcomes. Addressing challenges such as drug resistance, targeting GBM stem cells, and crossing the blood–brain barrier is essential for improving treatment efficacy. While current treatments offer limited benefits, emerging strategies such as immunotherapies, precision medicine, and drug repurposing show significant potential. Technologies like liquid biopsies, AI-powered diagnostics, and nanotechnology could help overcome obstacles like the blood–brain barrier and GBM stem cells. Ongoing research into combination therapies, targeted drug delivery, and personalized treatments is crucial. Collaborative efforts and robust clinical trials are necessary to translate these innovations into effective therapies, offering hope for improved survival and quality of life for GBM patients.

## Methodology

A rigorous systematic review of the literature was carried out using the PubMed database to identify studies published between 2008 and 2024. The search included the following keywords: “glioblastoma multiforme,” “drug repurposing,” “anticancer therapies,” “tumor microenvironment,” and “targeted therapy.” To ensure comprehensive results, the search also included synonyms and related terms for each keyword, such as “GBM,” “glioma,” “cancer therapy,” and “tumor microenvironment interactions.” Additional searches incorporated terms related to specific repurposed drugs and their mechanisms of action. Inclusion of Boolean operators (AND, OR) and filters for study types such as randomized controlled trials, cohort studies, and preclinical studies was applied to narrow results to the most relevant articles. To maintain consistency and interpretability, only studies published in English were considered.

### Inclusion and exclusion criteria, and risk of bias

This review includes studies on repurposed drugs with anticancer effects on glioblastoma (GBM), focusing on mechanisms such as apoptosis, autophagy, resistance pathways, and TME interactions. Studies employing human GBM cell lines, animal models, or clinical data were considered, particularly those addressing critical pathways such as the blood–brain barrier (BBB), glioblastoma stem cells (GSCs), and drug resistance. Research on advanced diagnostics (e.g., liquid biopsy, next-generation sequencing) and novel therapies, including targeted treatments, combination regimens, and immunotherapies, was also included. Only peer-reviewed, English-language studies published from 2008 to 2024 were considered.

Studies were excluded if they were irrelevant to GBM, drug repurposing, or advanced therapies; did not address molecular mechanisms or clinical applications; lacked experimental evidence or clear conclusions on GBM treatment efficacy; or were purely theoretical without empirical validation. Additionally, studies with significant biases, poor controls, reproducibility issues, or those not focused on repurposed drugs for GBM were excluded.

To minimize bias and ensure methodological rigor, a systematic approach was employed to prioritize experimental and clinical studies, while excluding unsupported or irrelevant research. Bias was minimized by using a diverse set of data sources and avoiding a preference for positive findings. Methodological rigor was ensured through critical evaluation of study controls, statistical analyses, and reproducibility. Funding bias was mitigated by excluding studies with potential conflicts of interest. However, the focus on English-language, peer-reviewed articles introduces potential language and information biases. Variability in study methodologies, particularly reliance on preclinical data for some repurposed drugs, may limit conclusions regarding clinical efficacy. Despite efforts to reduce bias, these limitations should be considered. Future reviews should explore ongoing or unpublished clinical trials to enhance comprehensiveness.

## Introduction

Glioblastoma (GBM) is a formidable and relentless Grade IV brain tumor, as classified by the World Health Organization (WHO) [1]. It is characterized by rapid progression, extensive infiltration, and resistance to therapy, making it one of the most challenging and lethal malignancies to treat. GBM typically arises in the frontal or temporal lobes and is marked by rapid cell division, abnormal blood vessel growth, and central necrosis [2]. Despite advancements in surgical techniques, radiation, and chemotherapy, the prognosis remains poor, underscoring the urgent need for novel treatment strategies. The development of gliomas is driven by complex genetic and epigenetic alterations [3], contributing to their heterogeneity and therapeutic resistance. Despite significant advances in understanding its molecular mechanisms, the prognosis for GBM remains poor, with a median survival rate of just 14–16 months [4]. The global incidence of GBM is 3.22 cases per 100,000 people, with a higher prevalence in males. Resistance to current treatments is a major hurdle, making GBM a leading cause of death and disability worldwide [4].

Given the poor prognosis and the difficulty in overcoming treatment resistance, there is an urgent need for more effective therapies. Traditional drug development is both time-consuming and costly, typically involves preclinical research followed by clinical trials to assess a drug's efficacy, toxicity, pharmacokinetics, and pharmacodynamics. Promising candidates move through Phase I–III trials to confirm safety and effectiveness, a process that usually takes 10–15 years and costs $1–2 billion. Unfortunately, fewer than 1% of these candidates make it to market. In response to these challenges, drug repurposing has become an increasingly appealing strategy in oncology. By leveraging the existing safety and dosage data for approved drugs, this approach allows for faster, more cost-effective development of new anticancer treatments. This strategy is especially important for GBM, where traditional therapies have shown limited effectiveness, and exploring alternative treatments may offer the best opportunity to improve patient outcomes [5].

This review highlights emerging therapeutic strategies for GBM, with a particular focus on drug repurposing. By leveraging approved non-oncological drugs, such as antidepressants, antipsychotics, anti-inflammatory agents, and metabolic modulators, researchers can explore novel mechanisms of action while benefiting from established safety profiles. While this review emphasizes non-oncological drug repurposing, the investigation of oncological drugs for GBM treatment also remains an active area of research. Beyond drug repurposing, advances in precision medicine and technological innovations, such as CRISPR and personalized treatment strategies, hold promise for more effective, tailored therapies. By integrating these approaches, researchers can accelerate the development of new treatment options, potentially improving patient outcomes and quality of life.

## Pathophysiology, diagnosis, and classification of glioblastoma

**Fig. 1:**
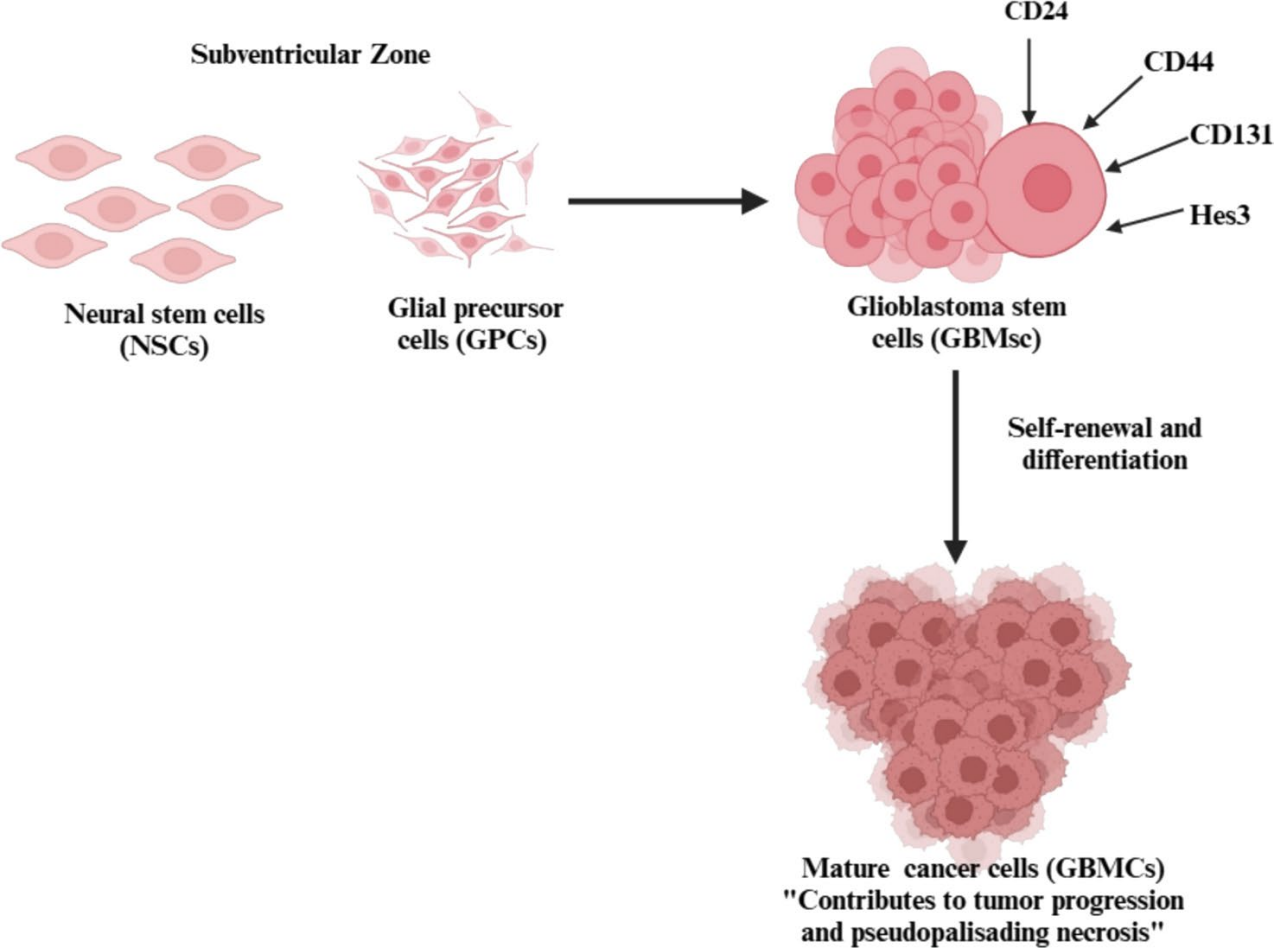
Origin of glioblastoma. Abbreviations: NSCs: neural stem cell; GPCs: glial precursor cells; GBMCs: glioblastomas

GBM is believed to arise from neural stem cells (NSCs) or glial precursor cells in the brain’s subventricular zone [6]. A hallmark of GBM is the presence of GSCs, which contribute to the tumor's resistance to treatment and drive its growth. These cells, which are marked by molecules such as CD24, CD44, CD133, and Hes3 [7], have the ability to self-renew and differentiate into more mature cancer cells, aiding in tumor progression and the formation of pseudopalisading necrosis (Fig. 1). An alternative hypothesis, the glial dedifferentiation theory, suggests that mature glial cells, such as astrocytes and oligodendrocytes, can revert to a stem-like state following oncogenic mutations, contributing to GBM initiation and progression. There are mutations in TP53, phosphatase and tensin homolog (PTEN), and epidermal growth factor receptor (EGFR). EGFR amplification has been shown to induce cellular reprogramming, enabling differentiated glial cells to regain proliferative and tumorigenic properties. Supporting evidence includes mesenchymal GBM subtypes exhibiting gene expression patterns similar to reactive astrocytes and experimental models demonstrating astrocyte reprogramming into GSCs under oncogenic stress. However, direct lineage-tracing evidence of glial dedifferentiation in vivo remains limited, making this theory less widely accepted than the SVZ neural stem/progenitor cell origin hypothesis. Nevertheless, both mechanisms may contribute to GBM heterogeneity and evolution, suggesting that multiple origins could be involved in tumor development.

The intricate biology of GBM, including its rapid growth, genetic diversity, and mechanisms of resistance, presents significant obstacles to treatment. These complexities underscore the need for novel therapeutic strategies, like drug repurposing, to target multiple pathways and overcome resistance.

**Fig. 2:**
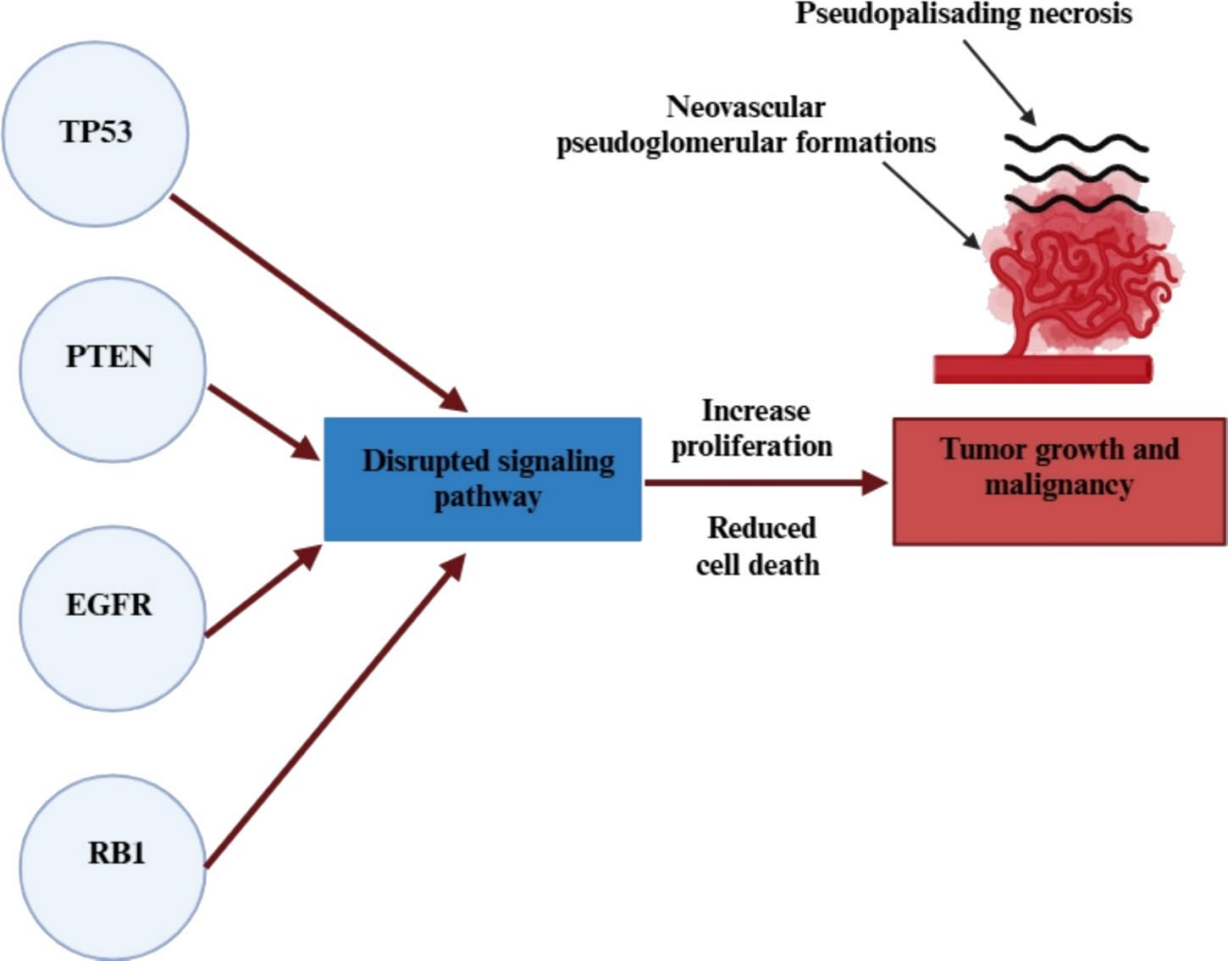
Key mutations of glioblastom. Abbreviations: TP53: tumor protein p53; PTEN: phosphatase and tensin homolog; EGFR: epidermal growth factor receptor; RB1: retinoblastoma gene 1

Key mutations in important tumor suppressor genes like TP53, PTEN, EGFR, and RB1 disrupt crucial signaling pathways, providing a growth advantage to the tumor and facilitating its malignant behavior [8]. Histologically, GBM is characterized by pseudopalisading necrosis and neovascular pseudoglomerular structures, which are driven by the secretion of growth factors [9] (Fig. 2).

The TME plays a pivotal role in GBM progression and treatment resistance. Reactive astrocytes, for example, foster tumor growth and invasion by transferring mitochondria to GBM cells [10]. Neurons also form electrical connections with GBM cells, altering neuronal networks to support tumor growth and spread [11]. Oligodendrocytes contribute to GBM by assisting with cell migration, blood vessel formation, and the establishment of glial stem cell niches at the tumor borders [12].

**Fig. 3:**
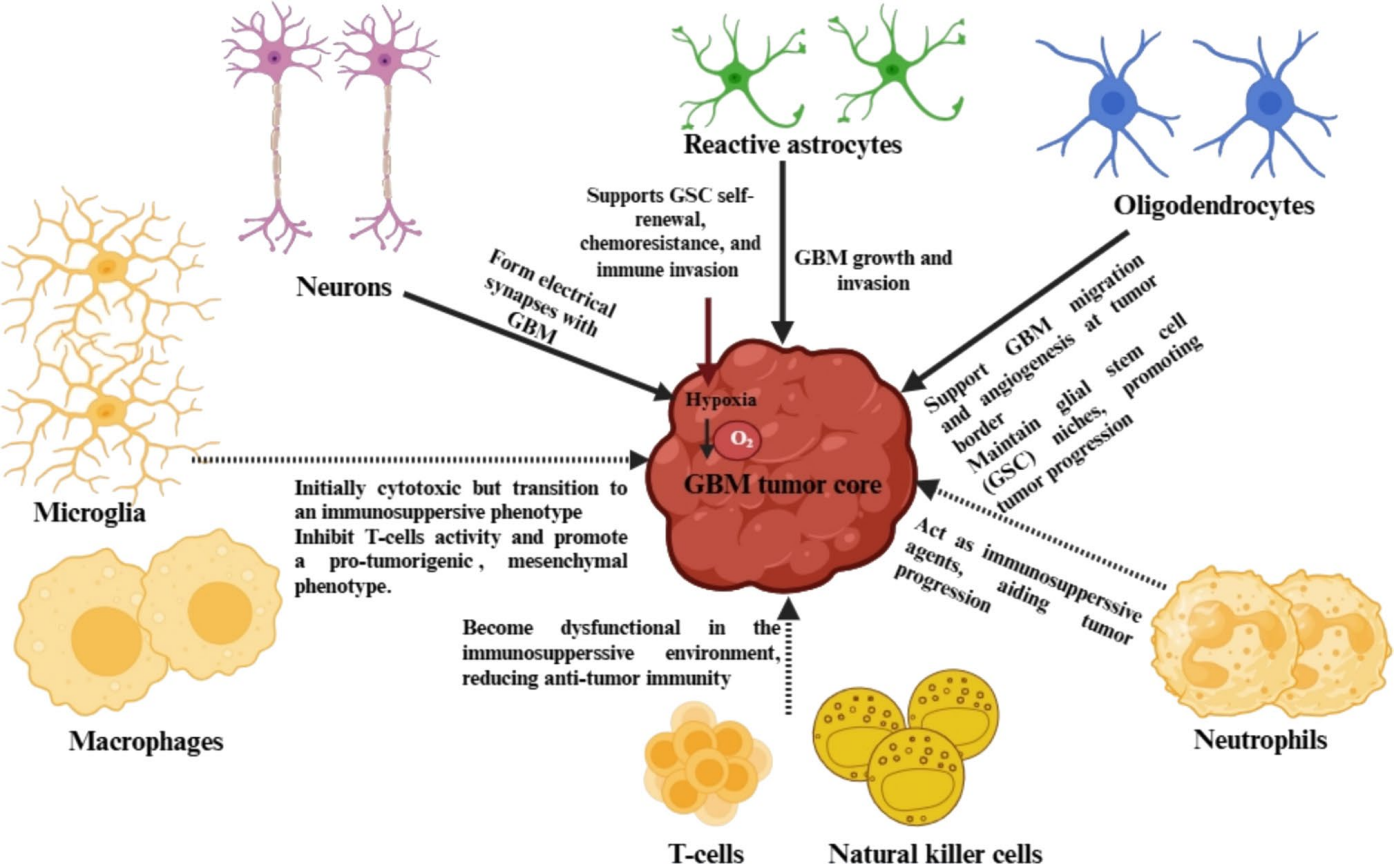
Tumor microenvironment of glioblastoma. Abbreviations: GBM: glioblastoma; GSC: glioblastoma stem cell

Immune cells are also crucial within the TME. While microglia and macrophages initially display cytotoxic activity, they eventually adopt an immunosuppressive phenotype, which inhibits T-cell function and promotes a tumor-friendly environment [13]. Neutrophils play a similar role in immune suppression [14], while natural killer (NK) cells and tumor-infiltrating lymphocytes become ineffective in this immunosuppressive setting [14] (Fig. 3).

Hypoxia is another critical factor in GBM pathophysiology. It drives GSC self-renewal, chemoresistance, and immune evasion [15]. Furthermore, metabolic reprogramming, particularly the increase in lactic acid production, supports tumor growth and suppresses immune responses against the tumor [16]. Tumor and stromal cells also release exosomes that alter cell behavior, contributing further to resistance [17] (Fig. 3). Understanding how angiogenesis and the BBB impact treatment delivery is vital for advancing therapeutic strategies, as these factors play a key role in the effectiveness of GBM treatments.

### Angiogenesis and the blood–brain barrier

**Fig. 4:**
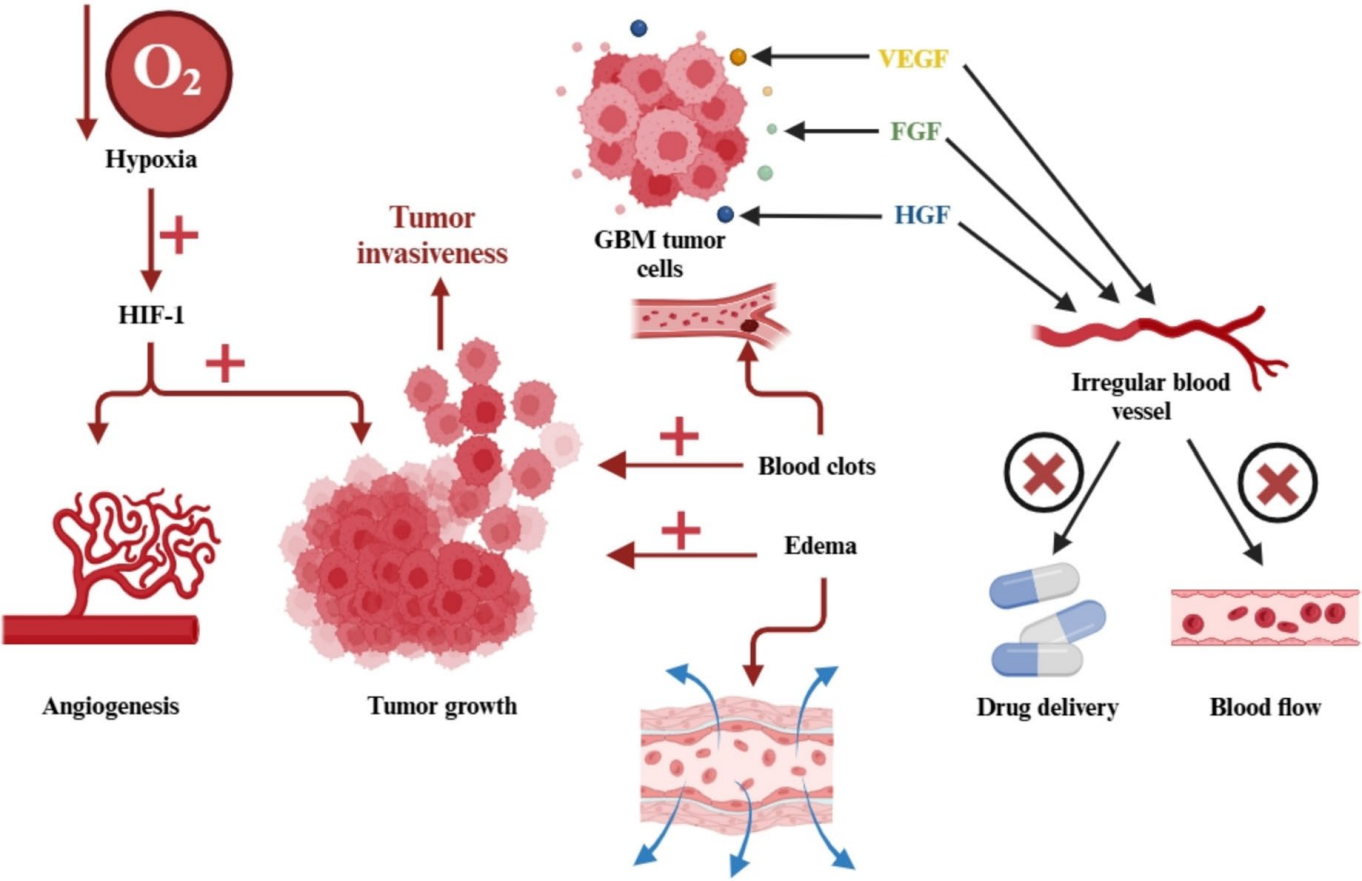
Tumor biology in glioblastoma: angiogenesis, invasion, hypoxia, and edema. Abbreviations: VEGF: vascular endothelial growth factor; FGF: fibroblast growth factor; HGF: hepatocyte growth factor; HIF-1: hypoxia-inducible factor-1

Angiogenesis plays a crucial role in the progression of GBM, primarily driven by the release of pro-angiogenic factors such as vascular endothelial growth factor (VEGF), fibroblast growth factor, and hepatocyte growth factor [18]. However, the newly formed blood vessels are often disorganized, which impairs blood flow and complicates the delivery of therapeutic drugs [19]. Hypoxia, resulting from high cell density and blocked blood vessels, leads to the creation of a necrotic core within the tumor. This oxygen-deprived environment contributes to therapy resistance, particularly because conventional chemotherapy relies on oxygen to generate reactive oxygen species (ROS), which are crucial for cancer cell destruction. In response to hypoxia, GBM cells increase the production of hypoxia-inducible factor-1 (HIF-1), which boosts angiogenesis through VEGF upregulation and enhances tumor invasion [20] (Fig. 4).

The BBB adds another layer of complexity to effective GBM treatment. This barrier regulates the exchange of substances between the bloodstream and the central nervous system (CNS), playing a key role in maintaining CNS homeostasis. The BBB consists of endothelial cells, basement membranes, pericytes, and astrocytes, and its selective permeability is controlled by tight junctions between endothelial cells and high transendothelial electrical resistance (TEER). These mechanisms restrict the passage of water-soluble and polar molecules, while allowing small, lipophilic molecules like oxygen and carbon dioxide to pass freely. Nutrient transporters also regulate the influx of essential compounds such as glucose and amino acids, as well as the efflux of harmful substances, including many drugs [21].

This selective permeability presents major challenges for GBM treatment, as most chemotherapy drugs are large, hydrophilic molecules that struggle to cross the BBB. GBM cells themselves worsen this issue in multiple ways. For instance, they overproduce VEGF, leading to the formation of immature, leaky blood vessels that weaken the tight junctions in the BBB, increasing its permeability and hindering drug delivery. Additionally, GBM cells invade surrounding brain tissue, triggering inflammation that further disrupts the BBB. The release of matrix metalloproteinases by GBM cells degrades the extracellular matrix and vascular basement membrane, further compromising the BBB’s integrity [22].

**Fig. 5:**
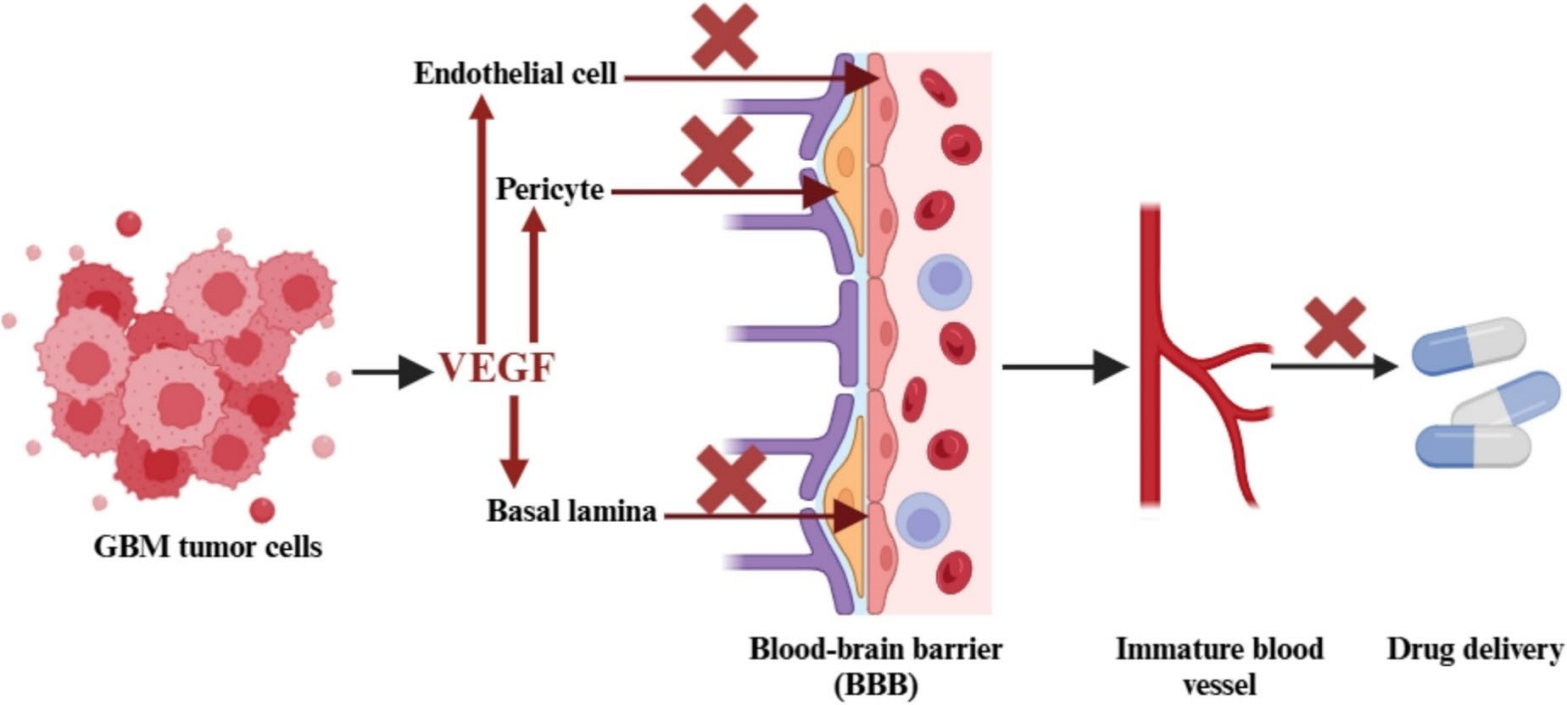
VEGF-mediated blood vessel formation and blood–brain barrier disruption in glioblastoma: implications for drug delivery. Abbreviations: BBB: blood–brain barrier; VEGF: vascular endothelial growth factor

Moreover, GBM cells alter the function of pericytes, which are vital for BBB stability and maintaining vascular tone, further weakening the barrier and promoting tumor growth and invasion. The BBB is not uniform throughout the tumor—some areas may exhibit a compromised barrier that allows limited drug penetration, while other regions may remain intact, leading to incomplete treatment and eventual tumor recurrence (Fig. 5). Adding to the difficulty, GBM cells often overexpress drug efflux pumps like P-glycoprotein (P-gp), which limits the uptake of therapeutic agents and reduces the effectiveness of treatment. P-gp is overexpressed in the endothelial cells of the BBB, making it even more challenging for drugs to penetrate the brain. In GBM cells, P-gp overexpression plays a significant role in resistance to drugs such as temozolomide (TMZ) [23].

### Tumor acidosis

Tumor acidosis plays a vital role in shaping the TME and has a significant impact on the effectiveness of therapies, particularly chemotherapy and immunotherapy. The acidic conditions in the TME contribute to treatment resistance by enhancing markers associated with glioma stem cells (GSCs), promoting tumor growth through the upregulation of HIF-2α-regulated angiogenic factors, and increasing autophagic activity, which supports the maintenance and aggressiveness of GSCs. Moreover, acidosis activates cathepsin L, an enzyme that converts plasminogen into plasmin, which in turn degrades extracellular matrix proteins and activates matrix metalloproteinases, facilitating tumor invasion [24].

From a therapeutic standpoint, tumor acidosis hinders the effectiveness of chemotherapy, especially for weak base drugs like doxorubicin and vincristine. The acidic environment induces ion trapping, which prevents these drugs from being absorbed properly and enhances the activity of P-glycoprotein (P-gp), a drug efflux pump, further diminishing their therapeutic efficacy. Acidosis also impairs immune responses, particularly by inhibiting the function of CD8 + T-cells, reducing cytokine production, receptor expression, and overall immune signaling, all of which hinder anti-tumor immunity. Furthermore, acidosis suppresses the effector functions of T-cells and monocytes, increases the recruitment of myeloid-derived suppressor cells (MDSCs), and reduces the cytotoxic activity of natural killer (NK) and NKT cells, all of which contribute to resistance against immunotherapy [25].

### Glutathione and therapy resistance in glioblastoma

Elevated levels of glutathione in GBM play a critical role in reducing oxidative stress, thereby supporting disease progression and promoting resistance to reactive oxygen species (ROS)-dependent chemotherapeutics. Resistant GBM cells exhibit higher glutathione levels and lower ROS levels compared to TMZ-sensitive cells, further underscoring its role in mediating therapy resistance. By neutralizing ROS, glutathione contributes to a cellular environment that enhances tumor survival and limits the effectiveness of treatments that rely on ROS to induce cancer cell death. Targeting the glutathione pathway presents a promising avenue for future GBM therapies, particularly within the context of drug repurposing. Repurposed drugs that modulate glutathione levels or its associated pathways could potentially reverse or reduce the resistance GBM exhibits to conventional therapies, offering new therapeutic strategies [26].

### Clinical features

**Fig. 6:**
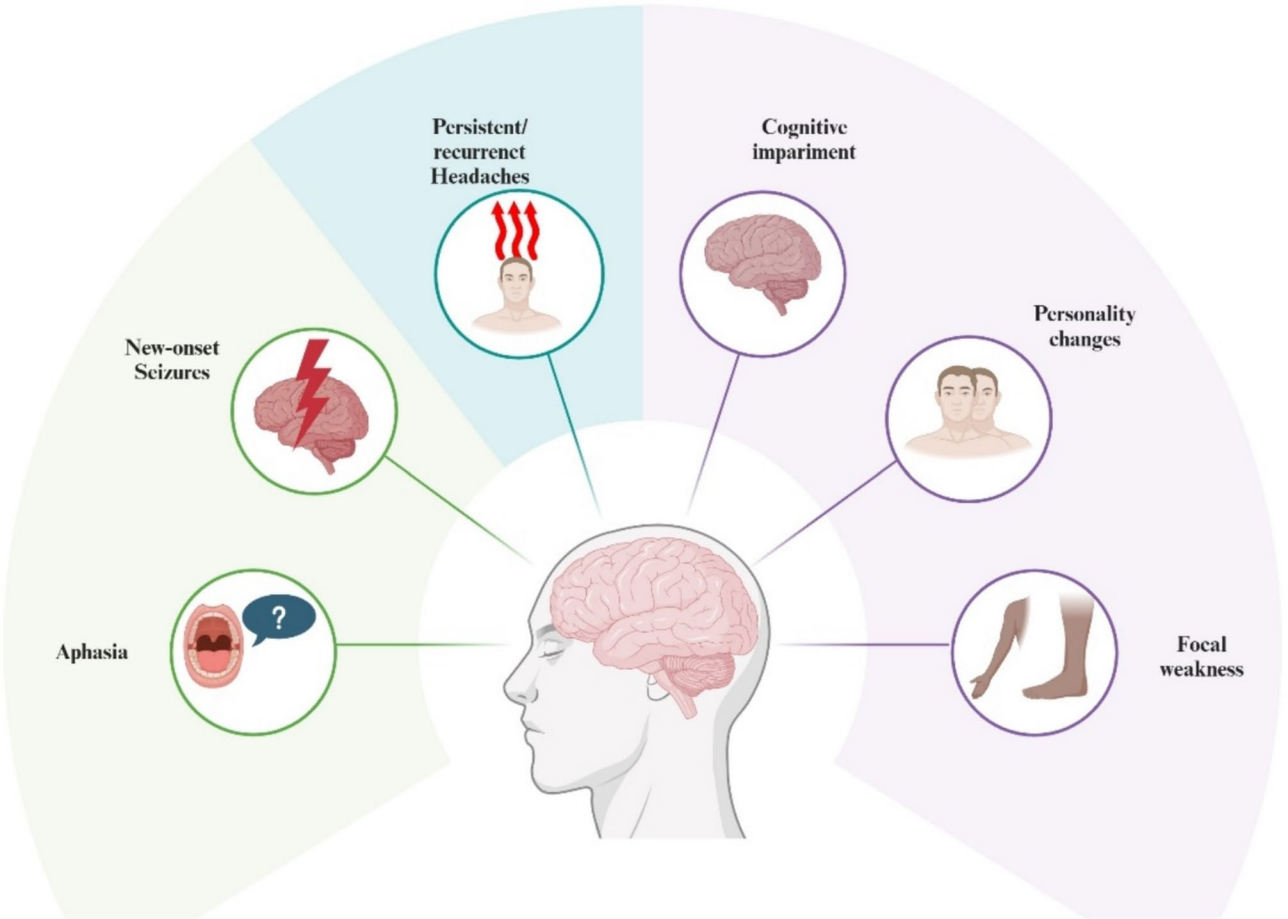
Clinical features of glioblastoma: early signs and neurological manifestations

The early symptoms of GBM are often vague and non-specific, making timely diagnosis and intervention challenging. As a result, the disease is frequently not detected until it reaches more advanced stages. Common early signs include persistent or recurrent headaches, which may intensify with activities such as coughing or changes in posture. Neurological symptoms, such as focal weakness, cognitive decline, and personality changes, are also frequently observed [27]. The onset of new seizures, particularly in individuals over 20 years old, is another concerning indicator of GBM [28]. Aphasia, which refers to difficulties with speech and language, typically occurs when the tumor affects language-processing regions of the brain, such as Broca’s or Wernicke’s areas [29] (Fig. 6). The delayed appearance of these clinical symptoms underscores the difficulty of early detection and highlights the critical need for more effective diagnostic tools and timely treatments to improve patient outcomes and quality of life.

### Next-generation sequencing (NGS)

Sanger sequencing, the first commercially available DNA sequencing method [30], utilized 2′-deoxynucleotides (dNTPs) for DNA synthesis and 2′,3′-dideoxynucleotides (ddNTPs) to terminate the synthesis, generating DNA fragments of various lengths. These fragments were analyzed using high-resolution gel electrophoresis. The automation of Sanger sequencing with fluorescently labeled primers or ddNTPs facilitated major milestones, including the completion of the Human Genome Project. Despite its groundbreaking success, Sanger sequencing faced limitations such as high costs, long processing times, low throughput, and inefficiency, which led to the development of next-generation sequencing (NGS) [31].

Building on the principles of Sanger sequencing, NGS uses polymerases, modified nucleotides, and fluorescent detection to sequence DNA more efficiently [32]. Platforms like Illumina, Ion Torrent, and PacBio provide much higher throughput at lower costs. Illumina's "sequencing by synthesis," Ion Torrent's proton-release detection, and PacBio's single-molecule real-time (SMRT) sequencing each contribute to the advancements in NGS technology [33]. For example, NGS platforms can sequence around 5,000 Mb per day at a cost of $0.50/Mb, while Sanger sequencing is limited to approximately 6 Mb per day at $500/Mb [34].

Since its introduction in 2005, NGS has revolutionized genetic research, enabling extensive studies on genetic variations, RNA profiles, methylation patterns, and chromatin immunoprecipitation. It has significantly advanced genomic medicine, facilitating personalized treatments, improving diagnostics, and enhancing our understanding of genetic factors in health. In the context of GBM, NGS plays a crucial role in identifying key molecular markers, such as mutations and gene expression patterns, which may be targeted by existing drugs. This makes NGS particularly valuable for drug repurposing, as it allows for the identification of genetic profiles that match available therapies to the unique molecular characteristics of a patient's GBM. However, widespread adoption of NGS requires significant infrastructure, skilled personnel, and raises ethical concerns regarding data ownership and the secondary use of genetic information [35].

### Third-generation sequencing

Third-generation sequencing improves on traditional NGS by allowing for single-molecule long reads, which addresses many of the limitations of earlier technologies [36]. Techniques like PacBio’s SMRT sequencing and nanopore sequencing offer real-time sequencing with less need for extensive sample preparation [37]. In particular, nanopore sequencing uses an electric field to pull single-stranded DNA or RNA through tiny pores, converting the resulting electrical signals into sequence data [21]. This approach makes it possible to read long DNA strands, often spanning several kilobases, with ongoing improvements in nanopore technology helping to address challenges like translocation speed and the size of recognition regions [22].

These advances in sequencing are helping move personalized medicine forward by enabling more detailed gene analysis, full exome and genome sequencing, and RNA profiling. Nanopore sequencing, for example, has shown it can sequence an entire human genome using ultra-long reads (up to 882 kb), all while being faster and more affordable, even in areas with fewer resources. This technology is also being tested for cancer diagnostics, allowing for the rapid identification of mutations in genes like IDH1, IDH2, TP53, H3F3A, and TERT promoter, as well as the 1p/19q codeletions. Notably, nanopore sequencing can provide 0.1X genome coverage in just six hours, and it can even detect DNA methylation profiles without needing special DNA conversion methods like bisulfite treatment [22, 38].

For GBM, which is often difficult to diagnose early due to vague symptoms, third-generation sequencing could significantly improve diagnostic accuracy. With its ability to deliver real-time sequencing and quickly identify mutations, nanopore sequencing could help doctors spot key genetic changes, such as those in IDH1 or TP53, which are crucial in GBM. This fast, high-resolution sequencing could help reduce diagnostic delays, allowing for quicker and more personalized treatment decisions that could ultimately improve patient outcomes. However, despite its impressive speed, portability, and affordability, nanopore sequencing still faces challenges, such as the need for large amounts of DNA (~ 700 μg) and the optimization required when dealing with fragmented nucleic acids. Ongoing improvements in both the technology itself and the software used to analyze it are necessary to unlock its full potential for widespread clinical use [38].

### Genetics of glioblastoma

The Cancer Genome Atlas (TCGA) has provided valuable insights into the key genetic changes present in GBM, highlighting three major pathways: receptor tyrosine kinase (RTK)/rat sarcoma (RAS)/PI3K (found in 88% of cases), p53 (78%), and retinoblastoma (RB) (87%). Some of the most common mutations include changes in the EGFR (45%), PTEN (36%), cyclin-dependent kinase inhibitors 2A (CDKN2A) and 2B (CDKN2B) (52% each), TP53 (35%), and RB deletions (11%) [39]. While mutations in IDH are rare in primary GBM that show alterations in EGFR and PTEN, these mutations are more frequently observed in lower-grade gliomas and in those that progress to higher-grade tumors [40].

Glioblastomas are molecularly diverse, which makes them particularly challenging to treat. These tumors can be divided into four subtypes: classical, proneural, neural, and mesenchymal [41]. Thanks to advances in single-cell RNA sequencing (scRNA-seq), we now have a much clearer understanding of the tumor cells and the surrounding microenvironment, including important profiles of GSCs, macrophages, microglia, and gene expressions linked to tumor development. Additionally, next-generation sequencing (NGS) panels have improved the accuracy of diagnosing GBM, offering a more precise molecular profile [42].

However, the complexity within GBM, including their genetic diversity, complicates treatment options. Tumors are often made up of different subclones, meaning that a single biopsy might not fully represent the genetic variability of the tumor. Treatments like TMZ can induce mutations that may drive tumor progression further. The genetic complexity of GBM, including mutations in key pathways like EGFR, PTEN, and TP53, plays a crucial role in therapy resistance. These findings point to the potential of repurposing existing drugs to target these specific mutations, offering a promising strategy for overcoming resistance. Transcriptome analyses, which study the RNA profiles of tumors, can refine GBM signatures, improve diagnostic accuracy, predict how tumors will respond to treatment, and help assess patient outcomes [43].

### Epigenetics of glioblastoma

Epigenetic modifications, such as DNA methylation, histone modifications, and chromatin remodeling, play a crucial role in regulating gene expression without changing the DNA sequence itself [44]. Chromatin, which consists of DNA wrapped around histone proteins (H1, H2A, H2B, H3, and H4) [45], forms nucleosomes that are essential for controlling gene activity [46]. When histones are acetylated by enzymes called histone acetyltransferases (HATs), the DNA–histone interactions are loosened, promoting gene expression. Conversely, deacetylation by histone deacetylases (HDACs) silences gene activity [47]. In pediatric gliomas, mutations in the H3.3 histone, such as K27M and G34R/V, are common. In contrast, adult GBMs, as defined by the current WHO classification, are typically IDH-wildtype. IDH1-mutant gliomas are now classified separately as astrocytomas (Grades II-IV). IDH1 mutations lead to the accumulation of 2-hydroxyglutarate (2-HG), an oncometabolite that inhibits histone demethylases, thereby altering H3K27 and H3K36 methylation patterns and affecting gene expression [47]. Methylation of lysine residues on histones can mark chromatin regions as either active (K4, K36, K39) or inactive (K9, K27), thus influencing gene transcription [48]. Chromatin remodeling mechanisms, such as the SWI/SNF core complex and the BRG1 protein, also contribute to GBM progression by affecting glioma stem cell tumorigenicity [49].

DNA methylation, where methyl groups are added to cytosine at CpG sites, is crucial for regulating gene expression. This modification helps silence transposable elements, inactivate X-chromosome genes, and maintain genome stability. While adenine methylation is less studied in mammals, it plays important roles in other organisms. In GBM, global hypomethylation is found in about 80% of cases and promotes the activation of oncogenes, aiding tumor growth [50]. On the other hand, hypermethylation at specific gene loci silences tumor suppressor genes. Methylation of CpG islands typically correlates with reduced gene expression. Studies have identified specific methylation patterns with prognostic value. For instance, the 1p/19q co-deletion (Codel) is associated with better prognosis in oligodendrogliomas, while glioma-CpG island methylator phenotype (G-CIMP) classification further refines prognosis, with G-CIMP-high tumors generally linked to better outcomes and G-CIMP-low tumors associated with worse prognosis. One key epigenetic biomarker is the methylation of the MGMT promoter, which is the only known predictor of response to TMZ. When the MGMT promoter is methylated, the tumor is more sensitive to chemotherapy, significantly improving patient survival. For methylated cases, the median survival increases from 16 to 43 months, compared to 11–36 months for unmethylated cases [51].

These epigenetic changes present valuable opportunities for drug repurposing. Targeting HDACs or using DNA demethylating agents could provide new ways to overcome treatment resistance [52]. By understanding how existing drugs might influence these epigenetic pathways, we could improve the effectiveness of treatments and better tailor them to the specific epigenetic profile of each GBM tumor.

### Classification

**Fig. 7:**
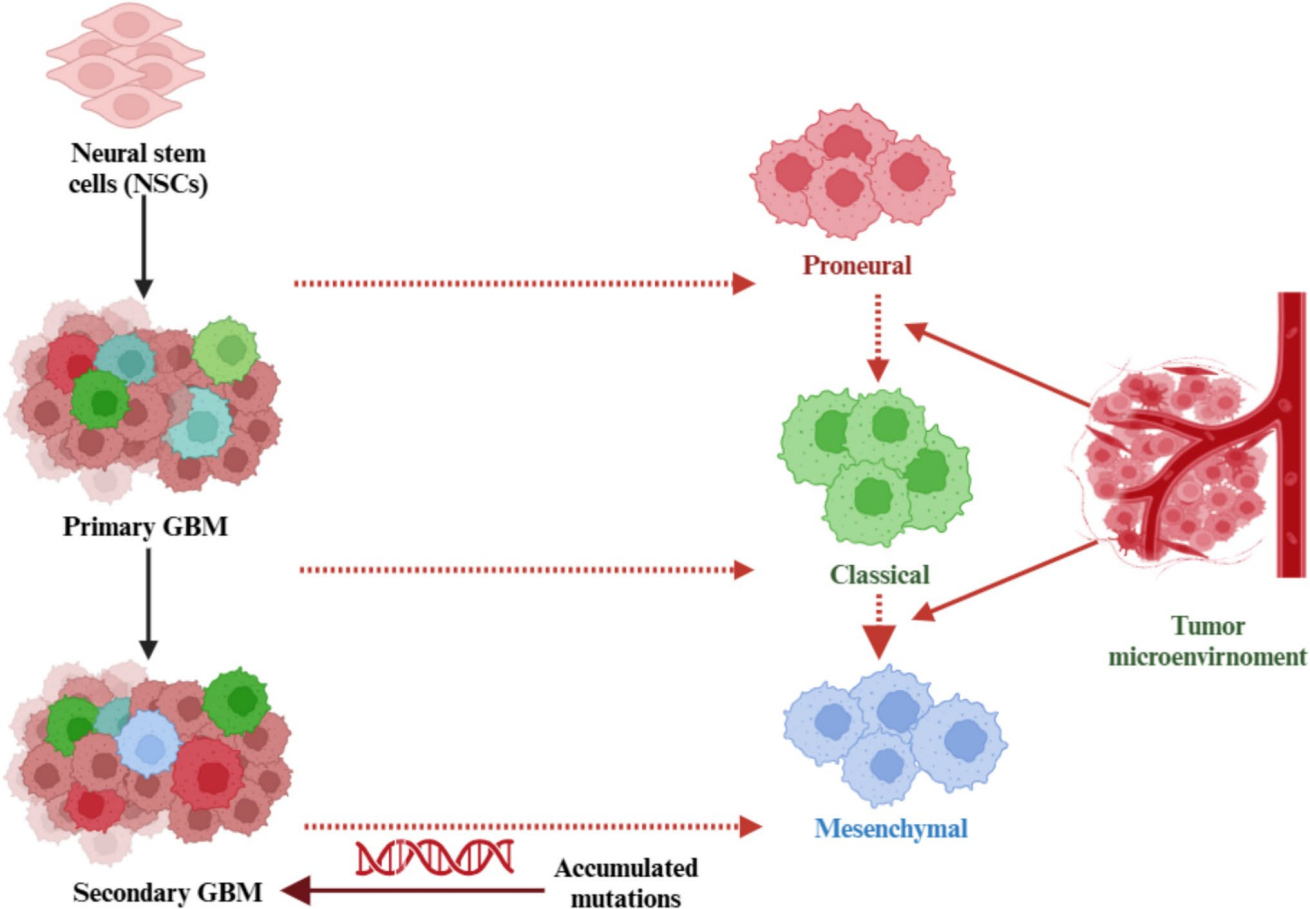
Molecular and phenotypic heterogeneity of glioblastoma: subtypes and origins. Abbreviations: NSCs: neural stem cells; GBM: glioblastoma

GBM is a highly heterogeneous tumor, meaning it varies greatly in terms of its genetic and phenotypic traits. It has several molecular subtypes, including proneural, classical, and mesenchymal types, each influenced by distinct genetic and phenotypic factors. According to the WHO 2021 classification, GBM is strictly defined as a Grade IV astrocytoma that is IDH-wildtype, reinforcing its aggressive nature, invasive properties, and treatment resistance. Astrocytoma, IDH-mutant (Grades II-IV), previously classified as “secondary GBM,” is now recognized as a separate tumor entity due to distinct molecular and clinical differences [53]. Moreover, GBM cells have the ability to switch between different phenotypes in response to changes in their surrounding environment, adding further complexity to treatment approaches [54] (Fig. 7).

**Fig. 8:**
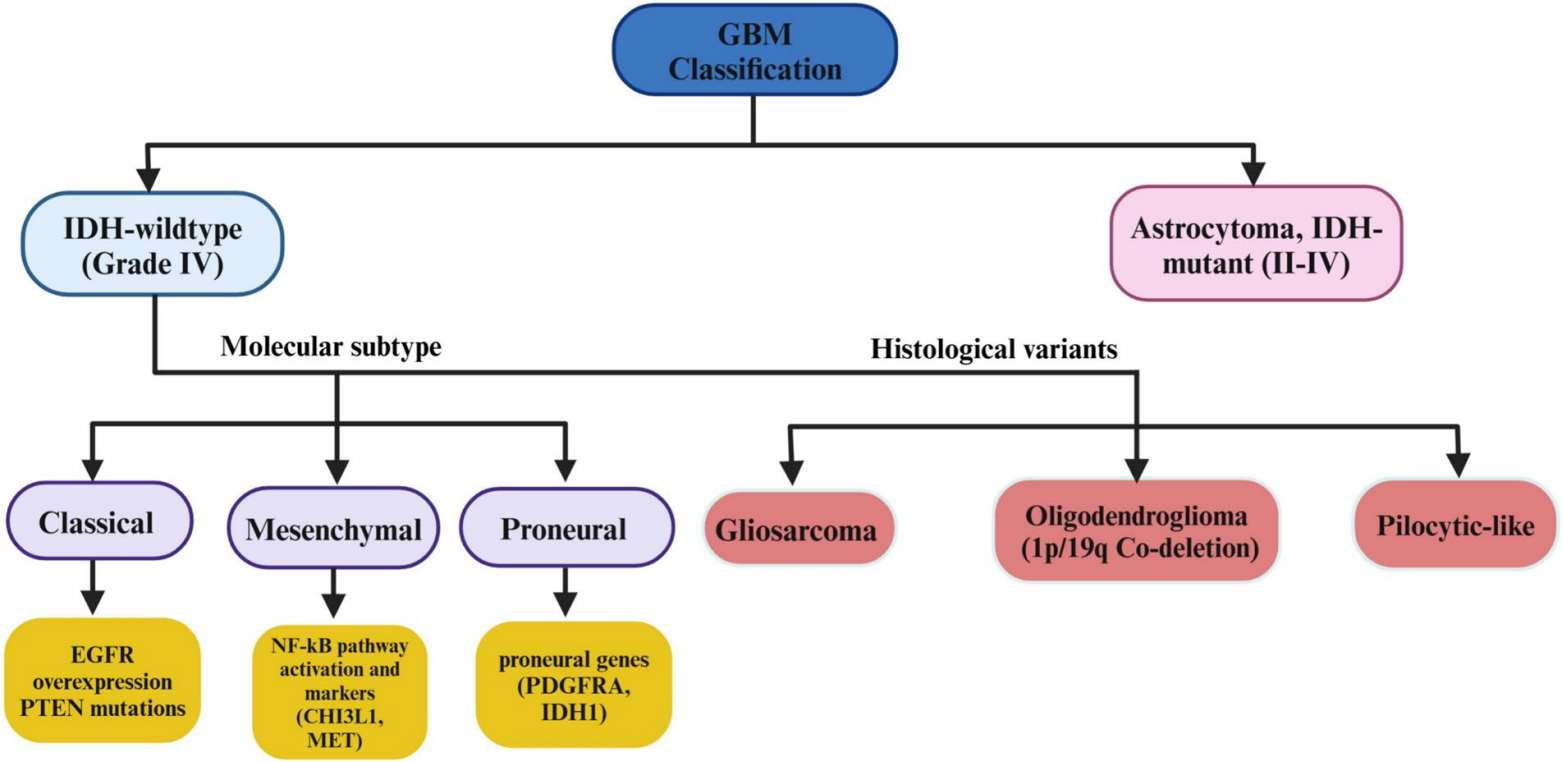
Comprehensive classification of glioblastoma: molecular, histological, and prognostic insights. Abbreviations: GBM: glioblastoma; EGFR: epidermal growth factor receptor; PTEN: phosphatase and tensin homolog; NF-κB: nuclear factor kappa-light-chain-enhancer of activated B-cells; CHI3L1: chitinase 3-like-1; MET: MET proto-oncogene, receptor tyrosine kinase; PDGFRA: platelet-derived growth factor receptor alpha; IDH: isocitrate dehydrogenase

Molecular classification systems, like the one developed by The Cancer Genome Atlas (TCGA), identify four main subtypes of GBM. Classical GBM is marked by the overexpression of the EGFR and mutations in the PTEN gene. Mesenchymal GBM, on the other hand, is characterized by the activation of the NF-κB pathway, increased necrosis, and inflammation, as well as the expression of mesenchymal markers such as CHI3L1 and MET, all of which contribute to a poorer prognosis. Proneural GBM is associated with the expression of genes like PDGFRA and IDH1, while neural GBM expresses proteins related to neural activity, such as NEFL and SLC1A3, although its clinical significance is still not well understood [55]. However, subsequent studies revealed that the neural subtype largely represented contamination from normal neuronal cells rather than a distinct tumor intrinsic classification. As a result, the neural subtype was removed, and the classification was refined into three main subtypes: classical, mesenchymal, and proneural (Fig. 8).

Beyond these molecular subtypes, GBM can also exhibit distinct histological variations. Some glioblastomas may present with histological features that resemble oligodendrogliomas; however, the defining 1p/19q co-deletion is exclusively observed in IDH-mutant, Grade II or III oligodendrogliomas and is not a feature of GBM (IDH-wildtype), as per the current WHO classification. Similarly, certain GBMs may display characteristics reminiscent of pilocytic astrocytoma, further complicating differential diagnosis. A rare histological variant of GBM, known as gliosarcoma, contains a sarcomatous component and is associated with a particularly poor prognosis. Importantly, IDH mutations are no longer considered part of GBM classification under the updated WHO guidelines. Instead, IDH-mutant Grade IV gliomas are now classified separately as astrocytoma, IDH-mutant (Grade II-IV), reflecting their distinct molecular and clinical behavior. This distinction is critical, as IDH-mutant astrocytomas generally have a better prognosis compared to IDH-wildtype GBM, reinforcing the importance of IDH status as a key biomarker for accurate diagnosis and personalized treatment planning [55] (Fig. 8).

By using these molecular and histological classifications, doctors can develop personalized treatment plans, including the possibility of repurposing existing drugs. Tailoring therapies to fit the specific genetic and phenotypic profile of each GBM subtype holds great potential for improving patient outcomes and overcoming the challenge of treatment resistance.

Beyond transcriptomic subtyping, a growing area of research has focused on the TME and its role in GBM progression and therapeutic response. A recent classification proposed by White et al. introduces a TME-based model for IDH-wildtype GBM, categorizing tumors into TME-high, TME-med, and TME-low groups. This classification is particularly relevant for immunotherapy stratification, as TME-high tumors, which exhibit increased immune cell infiltration, may respond differently to immune checkpoint inhibitors compared to TME-low tumors. As GBM treatment strategies evolve, incorporating both transcriptomic and TME-based classifications may aid in precision medicine approaches and personalized treatment strategies.

## Advancements in therapeutic approaches

Treatment for GBM has evolved significantly over the years, progressing from standard methods like surgery, radiation, and TMZ to more innovative therapies. The cornerstone of GBM treatment remains the goal of achieving the maximal safe surgical removal of the tumor, followed by radiation therapy and chemotherapy with TMZ. One promising addition to the treatment arsenal is tumor-treating fields, which involve the use of low-intensity alternating electric fields and have been approved by the FDA. When used alongside traditional treatments, these fields have shown some benefit in terms of survival [55].

Targeted therapies aim to tackle specific genetic abnormalities in GBM cells, but they face challenges such as differences in how patients respond to treatment and the development of drug resistance. For instance, inhibitors of the EGFR, such as erlotinib and gefitinib, have failed to demonstrate significant clinical benefit in GBM. The reasons for these failures are multifactorial and remain incompletely understood, involving challenges such as receptor downregulation, limited BBB penetration, the development of resistance mechanisms, and tumor heterogeneity. These factors have limited the efficacy of EGFR-targeted therapies in GBM, underscoring the need for alternative therapeutic strategies [56].

IDH inhibitors, such as ivosidenib and vorasidenib, have shown promise in treating IDH-mutant gliomas. Notably, vorasidenib is now approved for the treatment of Grade II IDH1- or IDH2-mutant astrocytomas, marking a significant advancement in targeted therapy. However, the effectiveness of IDH inhibitors in high-grade gliomas remains an area of ongoing investigation, with clinical trials evaluating their potential impact on disease progression and patient outcomes [57].

Immunotherapy has become an exciting area of research, with treatments like checkpoint inhibitors (e.g., anti-PD-1, anti-CTLA-4), CAR-T-cell therapy, and oncolytic viruses (Ovs) showing potential. Ovs have shown promise as a novel therapeutic strategy for GBM. However, their effectiveness is challenged by the brain’s inherently immunosuppressive tumor microenvironment (TME) and the molecular heterogeneity of GBM, which can limit viral replication and immune activation. OVs selectively infect and lyze tumor cells while sparing normal tissue. While some genetically modified OVs are engineered to enhance tumor specificity and therapeutic efficacy, others are naturally oncotropic, meaning they inherently exhibit tumor-selective tropism without genetic modification. These viruses exert their anticancer effects through a combination of direct oncolysis, immune system activation, and modulation of the TME, making them a promising avenue for GBM treatment. Advancements in drug delivery, such as focused ultrasound (FUS) and nanoparticles, are helping to overcome the BBB and enhance treatment efficacy in GBM. FUS is particularly promising for its ability to transiently disrupt the BBB, enabling more effective drug penetration into tumors while minimizing systemic toxicity. By using low-intensity ultrasound waves with microbubbles, FUS temporarily increases BBB permeability, improving the delivery of chemotherapy, immunotherapy, and targeted therapies. This approach is currently being evaluated in clinical trials, further exploring its potential to optimize GBM treatment. Bevacizumab, an anti-VEGF therapy, has been shown to enhance the effects of radiation and chemotherapy, though its ability to extend survival has been limited [58].

Cancer vaccines are another avenue of treatment. These vaccines aim to stimulate the immune system to recognize and attack tumor cells. There are two main types: peptide vaccines, which contain tumor-associated or tumor-specific antigens (such as EGFRvIII or mutated IDH enzymes), and cell-based vaccines, such as dendritic cell (DC) vaccines. In DC vaccines, immune cells are taken from the patient, loaded with tumor antigens, and then re-infused to activate T-cells. While clinical trials of DC vaccines have shown some success, challenges remain with antigen variability and the immunosuppressive nature of GBM’s environment. Tumor cell vaccines, which involve using the patient’s own tumor cells or cells from a donor, aim to boost the immune system’s response but also face obstacles related to antigen recognition and the TME [59, 60].

Emerging technologies like CRISPR/Cas9 and nanocarriers are showing great promise. CRISPR/Cas9 allows for precise editing of genes, potentially targeting resistance-related genes such as MGMT and ALDH1A3 or modulating the TME [61]. Nanocarriers, such as liposomes, polymeric micelles, silica nanoparticles, and exosomes, are developed to improve drug delivery directly to GBM cells, with an added focus on targeting specific receptors. Notably, exosome-based drug delivery is emerging as a promising strategy, offering biocompatibility, the ability to cross the BBB, and reduced immunogenicity. These features make exosomes attractive candidates for delivering RNA-based therapies, small-molecule drugs, and gene-editing tools, further expanding the potential of nanomedicine in GBM treatment [62].

In recent years, single-cell RNA sequencing (scRNA-seq) has dramatically improved our understanding of GBM’s complexity by identifying molecular subclones that drive tumor growth and contribute to resistance to treatment [63]. Bioinformatics and machine learning are further enhancing GBM research, helping to identify novel biomarkers, molecular signatures, and potential therapeutic targets, which could pave the way for more personalized treatment strategies [64].

**Fig. 9:**
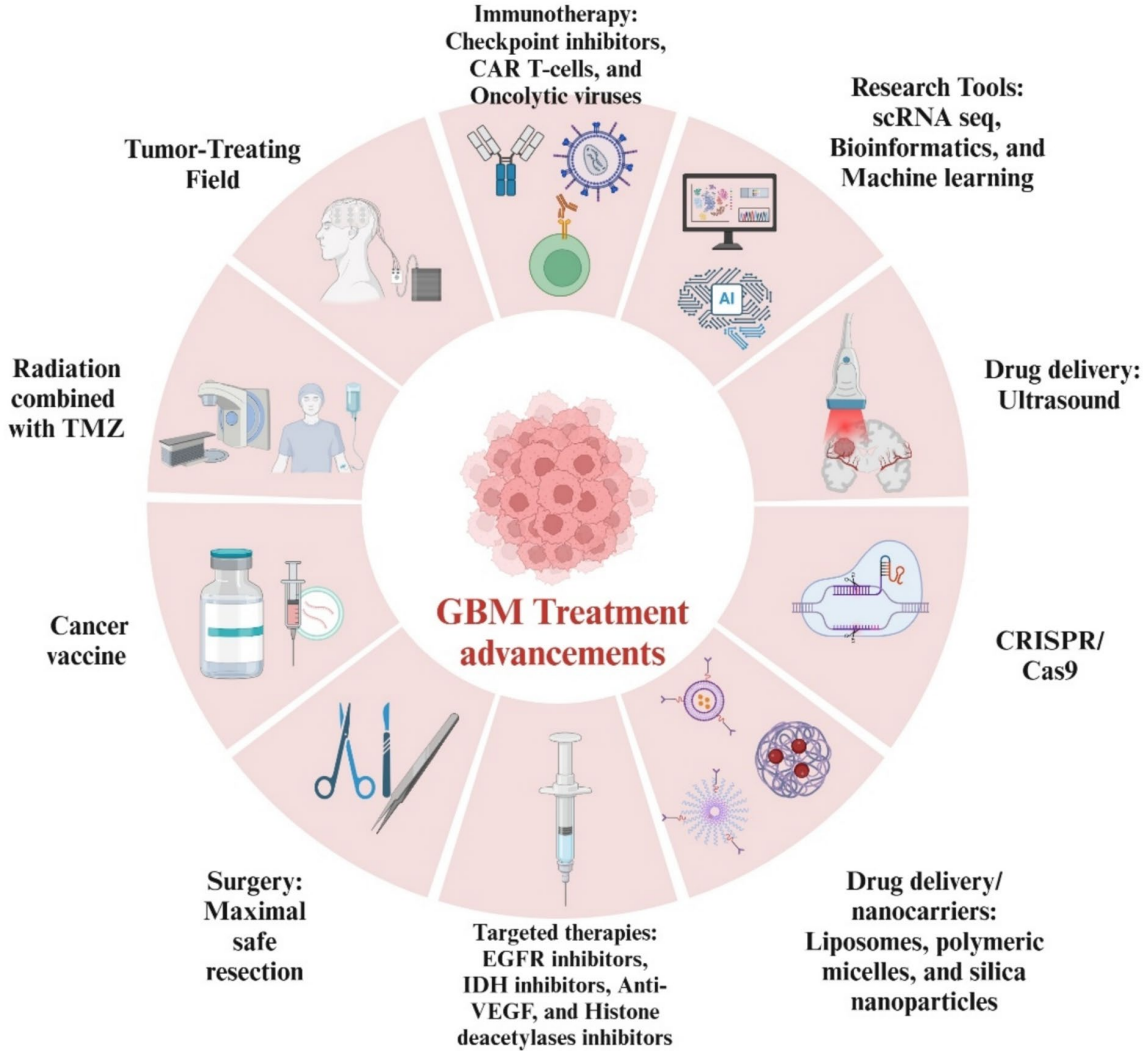
Advancements in glioblastoma therapy: from standard care to innovative strategies. Abbreviations: GBM: glioblastoma; TMZ: temozolomide; EGFR: epidermal growth factor receptor; IDH: isocitrate dehydrogenase; CAR-T: chimeric antigen receptor T-cell; CRISPR: clustered regularly interspaced short palindromic repeats; Cas9: CRISPR-associated protein 9; scRNA-seq: single-cell RNA sequencing

Lastly, epigenetic profiling is emerging as a promising tool in GBM treatment. Changes in histone acetylation, for example, could offer a new target for therapy. Histone deacetylase (HDAC) inhibitors, such as vorinostat, romidepsin, and valproic acid, are currently investigated for their potential to treat GBM. However, challenges remain in understanding how these drugs work, their impact on cellular signaling, and how to effectively deliver them across the BBB. Despite these challenges, these advances collectively provide hope for more effective, individualized treatments for GBM (Fig. 9).

### Multimodal and combination therapies

Combination therapy, which involves using two or more treatments with different mechanisms, has shown great promise in improving outcomes for patients with GBM. A key example is the combination of TMZ with radiotherapy, which is currently the standard treatment for GBM. This combination works well because TMZ, an alkylating agent that damages DNA, complements the effects of radiotherapy, which targets fast-growing tumor cells [65].

Efforts to enhance the effectiveness of TMZ continue, with several promising combinations. One approach pairs TMZ with MGMT inhibitors, which target the DNA repair enzyme MGMT and may help overcome TMZ-resistance. This combination is particularly relevant in patients with unmethylated MGMT promoters, where MGMT is actively expressed and counteracts TMZ-induced DNA damage. In contrast, in methylated MGMT tumors, where MGMT expression is silenced, TMZ remains more effective without the need for MGMT inhibition [66]. Other strategies include combining TMZ with inhibitors that target the PI3K/mTOR pathway, such as XL765, which has shown to increase cancer cell death in animal models of GBM [67]. Similarly, combining TMZ with bromo-domain inhibitors like JQ1 can promote DNA damage and help trigger cancer cell death [68]. Additionally, pairing TMZ with the anti-EGFR antibody nimotuzumab has shown potential for improving treatment effectiveness by targeting the EGFR mutation common in many GBM cases [69].

In addition to enhancing TMZ treatments, researchers are also focusing on targeting survival pathways in GBM cells to make treatments more effective. One example is the use of cyclopamine, a drug that blocks the Shh pathway, in combination with TMZ. This combination helps to prevent tumor recurrence [70]. Another promising approach uses γ-secretase inhibitors (GSIs) alongside radiation therapy, sensitizing GBM cells to radiation and improving the response to treatment [71]. Additionally, pairing TMZ with morphine has shown potential in overcoming drug resistance, specifically by blocking a mechanism (P-gp1) that prevents TMZ from entering tumor cells. This combination helps reduce tumor growth and improve the long-term success of treatment [72].

**Fig. 10:**
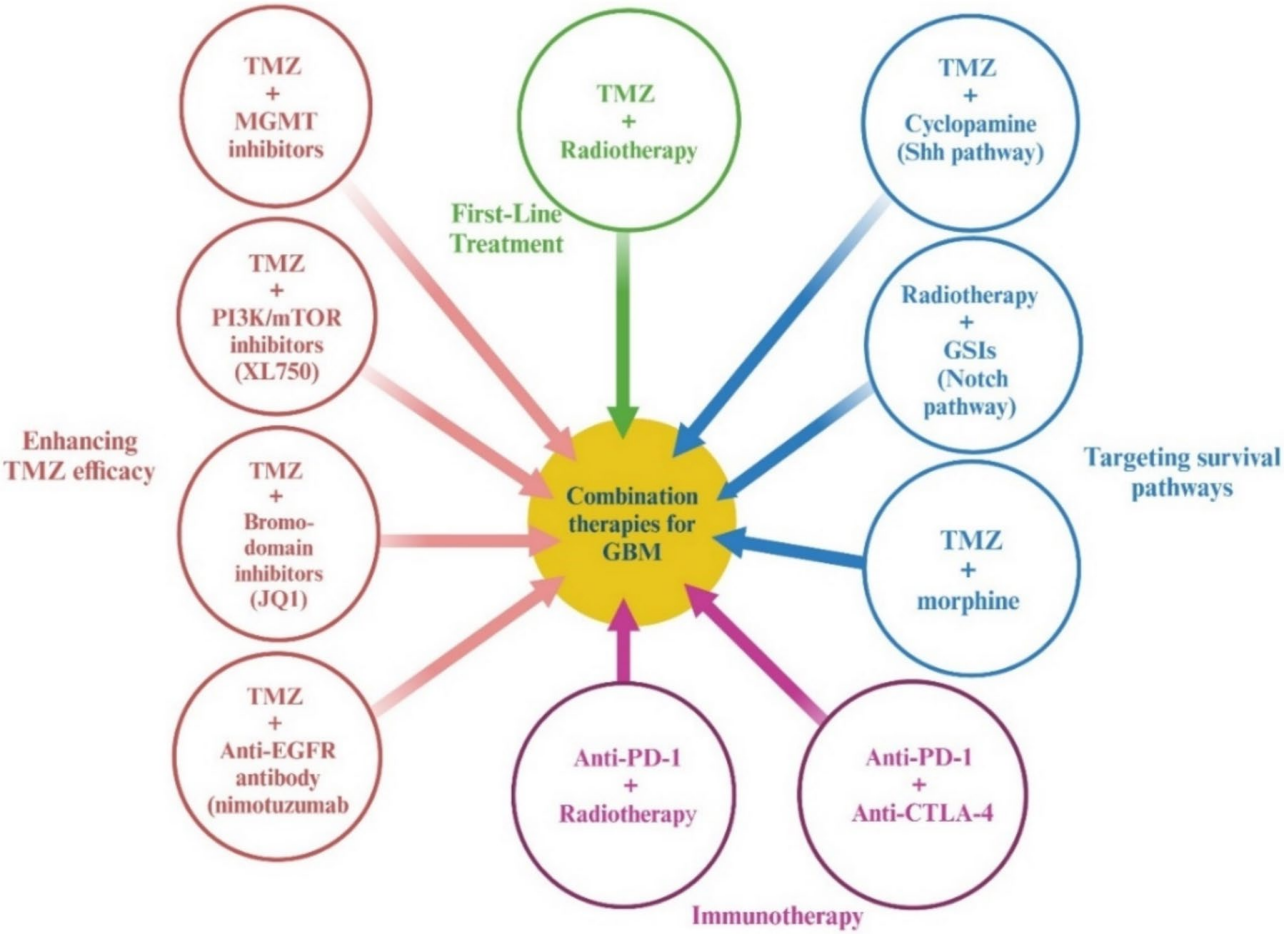
Multimodal approaches in glioblastoma: enhancing outcomes through combination therapies. Abbreviations: GBM: glioblastoma; TMZ: temozolomide; MGMT: O6-methylguanine-DNA methyltransferase; PI3K: phosphoinositide 3-kinase; mTOR: mechanistic target of rapamycin; EGFR: epidermal growth factor receptor; Shh: Sonic hedgehog; GSIs: γ-secretase inhibitors; PD-1: programmed death-1; CTLA-4: cytotoxic T-lymphocyte antigen

Beyond traditional treatments, combination therapy is also being explored in immunotherapy. One exciting strategy involves combining anti-PD-1 antibodies with other immunotherapies to enhance immune response in GBM. While early studies in animal models suggested potential benefits, clinical trials evaluating anti-PD-1 monotherapy, including Phase III trials and its upfront use with the Stupp protocol, have not demonstrated significant survival benefits. As a result, current research is focusing on combining anti-PD-1 therapy with other immunotherapeutic approaches, such as CTLA-4, tumor vaccines, or adoptive T-cell therapies, in an effort to improve treatment efficacy in GBM [73]. Another promising combination is using both anti-PD-1 and anti-CTLA-4 antibodies, which together enhance the immune response by reducing the number of immunosuppressive cells (MDSCs) in the TME, helping the immune system to target the tumor more effectively [74] (Fig. 10).

### Biomarkers and precision medicine

The molecular complexity of GBM underscores the need for reliable biomarkers that can improve diagnosis, prognosis, and the personalization of treatment. Established biomarkers, including IDH1, MGMT, and EGFR, provide crucial insights into the tumor's biology and are essential for guiding therapy decisions.

IDH1 mutations are predominantly found in lower-grade gliomas and IDH-mutant astrocytomas (Grades II-IV), which were previously referred to as secondary glioblastomas. However, under the current WHO classification, GBM is strictly defined as IDH-wildtype, and IDH-mutant high-grade gliomas are now classified separately as astrocytoma, IDH-mutant, Grades II-IV. These mutations are associated with a better prognosis and improved response to therapy, making IDH status a key molecular marker in the WHO classification system for gliomas [75, 76].

MGMT, an enzyme involved in repairing DNA damage, plays a key role in treatment effectiveness. When the MGMT gene promoter is methylated, it improves the tumor's response to chemotherapy with TMZ [77]. EGFR, frequently overexpressed or mutated in GBM, particularly in the EGFRvIII variant, is associated with aggressive tumor behavior and poor survival outcomes, although therapies targeting EGFR have had limited success [56]. Additionally, emerging biomarkers like ATRX and TP53 mutations are explored to refine prognosis and treatment strategies further [78].

Another set of important biomarkers includes mutations in the TERT promoter, which activate telomerase and promote telomere elongation, enabling sustained tumor cell growth. This makes TERT a crucial marker for prognosis and potential therapeutic targets [79]. VEGF, which is overexpressed in GBM, stimulates angiogenesis and helps form dense blood vessels that supply the tumor with nutrients and oxygen, contributing to rapid tumor growth and resistance to therapy. Similarly, the concurrent loss of CDKN2A and CDKN2B, which encode critical cell cycle regulators, contributes to uncontrolled cell division and tumor progression. This deletion leads to the loss of p16 (INK4a), which regulates Rb signaling; p14 (ARF), which modulates the p53 tumor suppressor pathway; and p15 (INK4b), which inhibits CDK4 activity, collectively promoting GBM proliferation and resistance to cell cycle control mechanisms [80]. NF1, another tumor suppressor gene, inhibits the Ras signaling pathway. Mutations in NF1 are linked with the mesenchymal subtype of GBM, which tends to be more invasive and associated with poorer survival [81].

Other markers like matrix metalloproteinases (MMPs), especially MMP-2 and MMP-9, play a critical role in GBM invasion by degrading the extracellular matrix, making them potential targets for limiting tumor progression [82]. The c-MET receptor, which is overexpressed in GBM, enhances cell proliferation and migration, positioning it as a valuable prognostic biomarker [83]. S100 proteins, such as S100B, are elevated in GBM and influence cell growth, offering potential for both diagnostic and therapeutic applications [84].

Liquid biopsy has emerged as a non-invasive method for monitoring GBM using biomarkers like circulating tumor cells (CTCs), extracellular vesicles (EVs), and cell-free nucleic acids. Liquid biopsy enables real-time treatment monitoring, early detection of recurrence, and differentiation between pseudoprogression and actual tumor progression. There are two primary strategies for liquid biopsy: one that detects tumor-specific materials like circulating tumor DNA (ctDNA), and another that analyzes biofluid components altered by GBM, such as immune cells. Both strategies significantly enhance diagnostic accuracy and enable more personalized treatment options [85].

CTCs, released from tumors through apoptosis or necrosis, provide valuable information about tumor size, mutations, and recurrence. Despite challenges posed by the BBB, ctDNA can help detect mutations in critical genes like TERT, MGMT, and EGFR, complementing invasive biopsy results. Cerebrospinal fluid (CSF) ctDNA has shown higher diagnostic accuracy than blood ctDNA, with advanced techniques like methylation-based PCR and next-generation sequencing (NGS) improving sensitivity [86].

EVs, including exosomes and microvesicles, carry nucleic acids, proteins, and metabolites that reflect the tumor's biology. EV markers such as EGFRvIII and PD-L1 show promise as diagnostic and prognostic tools. Elevated plasma EV levels correlate with tumor progression and recurrence, and techniques like Raman spectroscopy and flow cytometry are advancing the detection of EVs for clinical use [87].

Tumor-associated RNAs, including microRNAs (miRNAs) and circular RNAs (circRNAs), are stable and abundant biomarkers in GBM. Oncogenic miRNAs, like miR-10b, are elevated in GBM, while tumor suppressor miRNAs, such as miR-29a, decrease during tumor progression. Multi-RNA signatures, particularly combinations of miRNAs like miR-21 and miR-15b, have demonstrated superior diagnostic accuracy. Advances in RNA sequencing further support the development of reliable liquid biopsy assays for GBM [88].

**Fig. 11:**
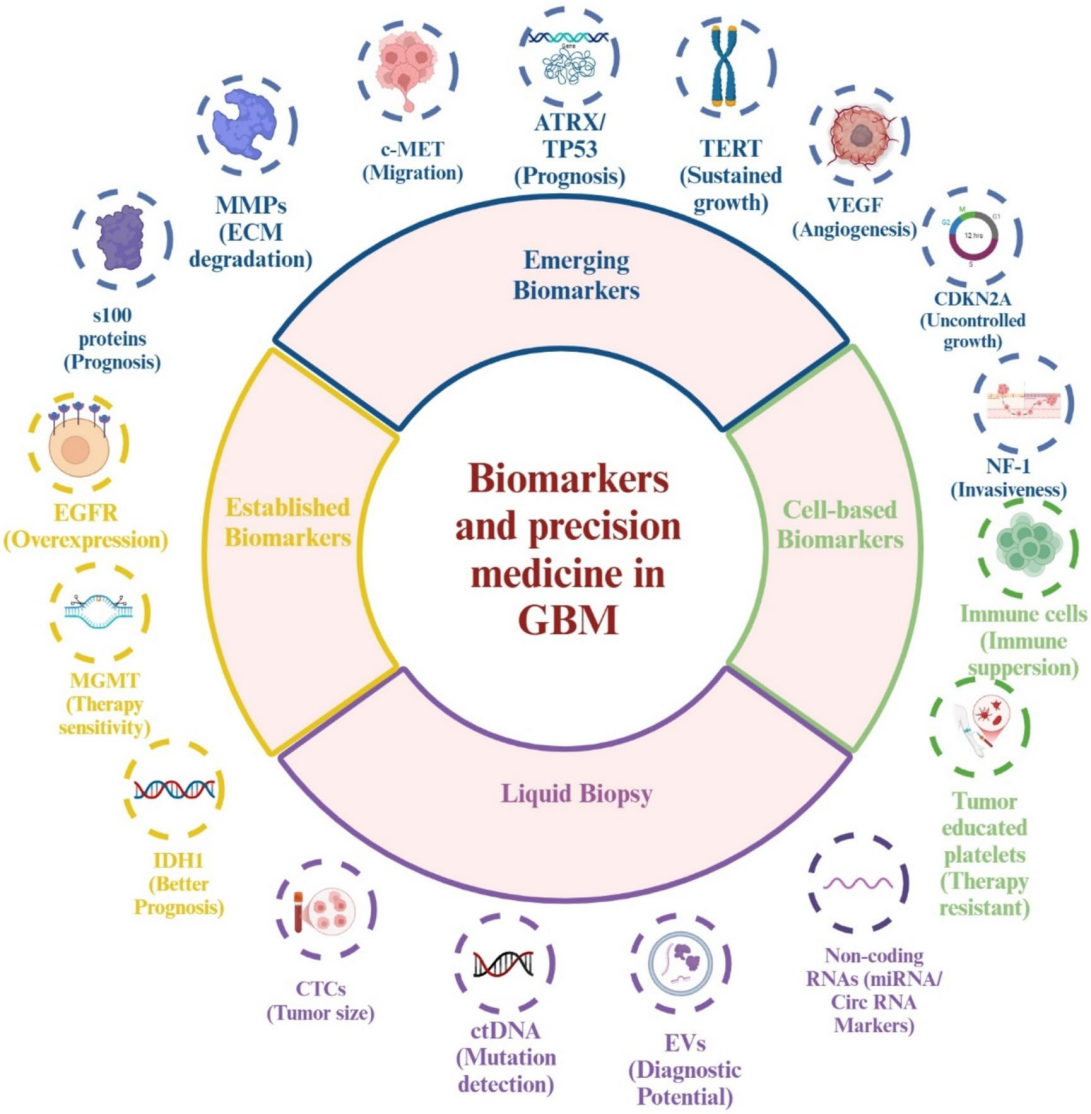
Biomarkers and precision medicine: unlocking insights into glioblastoma: diagnosis, prognosis, and treatment. Abbreviations: GBM: glioblastoma; IDH1: isocitrate dehydrogenase 1; MGMT: O6-methylguanine-DNA methyltransferase; EGFR: epidermal growth factor receptor; ATRX: alpha thalassemia/mental retardation syndrome X-linked; TP53: tumor protein P53; TERT: telomerase reverse transcriptase; VEGF: vascular endothelial growth factor; CDKN2A: cyclin-dependent kinase inhibitor 2A; p16INK4a: cyclin-dependent kinase inhibitor 2A, isoform p16; NF1: neurofibromin 1; MMP: matrix metalloproteinase; ECM: extracellular matrix; c-MET: MET proto-oncogene, receptor tyrosine kinase; S100B: S100 calcium binding protein B; CTCs: circulating tumor cells; EVs: extracellular vesicles; ctDNA: circulating tumor DNA; PD-L1: programmed death-ligand 1; miRNAs: MicroRNAs; circRNAs: circular RNAs

Finally, cell-based approaches that analyze circulating immune cells and CTCs provide additional insights. Although isolating CTCs remains difficult due to their low frequency, global blood analysis can overcome this limitation. GBM induces systemic immunosuppressive effects via monocytes, which differentiate into suppressive cells such as myeloid-derived suppressor cells (MDSCs) and M2 macrophages. Elevated levels of CD163 + monocytes and tumor-educated platelets, which express VEGFR1/2 and vWF, can serve as biomarkers for GBM progression and therapy resistance [89] (Fig. 11).

## Multi-omics in glioblastoma

Integrative omics approaches are significantly advancing our understanding of GBM by combining genomics, transcriptomics, epigenomics, and proteomics to provide a more comprehensive view of the disease. These studies reveal the intricate interactions between genetic alterations, cellular phenotypes, and the TME [90, 91]. A key insight from these approaches is the recognition of intratumoral heterogeneity, where a single tumor mass can harbor a diverse array of cellular states [92].

One of the most powerful tools in this area is single-cell RNA sequencing (scRNA-seq), which has been instrumental in identifying distinct cellular phenotypes in GBM. These phenotypes—such as astrocyte-like, oligodendrocyte precursor cell-like, and neural progenitor cell-like states—mirror both normal brain development and the genetic instability seen in GBM [91]. This ability to map out the cellular diversity within a tumor is critical in understanding the tumor's complex biology.

Multi-omics analyses, which integrate genomic, proteomic, and transcriptomic data, provide critical insights into how genetic mutations interact with cellular states and the TME, paving the way for more personalized and effective GBM treatments [93]. These studies have also uncovered key immune cell interactions within the tumor, identifying novel immunotherapy targets. A promising advancement in precision medicine is the use of ex vivo drug screening on patient-derived tumor cells, allowing for the identification of effective repurposed or novel therapeutic agents before clinical administration. This approach, currently being evaluated in clinical trials, provides real-time functional testing of drug efficacy, ensuring a more data-driven and individualized treatment strategy. By integrating multi-omics profiling with personalized drug screening, researchers aim to optimize therapeutic selection, improve treatment outcomes, and enhance patients' quality of life.

## Artificial intelligence in glioblastoma research and treatment

Artificial intelligence (AI) is transforming GBM diagnostics and treatment strategies. Machine learning (ML), a subset of AI, leverages large datasets to predict outcomes and enhance performance. Unlike traditional statistical models that analyze relationships between covariates and outcomes, ML uses data-driven approaches to make predictions. This technology is particularly valuable in genomics, where extensive genetic datasets generated through next-generation sequencing (NGS) are increasingly routine in biomedical research [94].

### Machine learning for disease diagnosis and therapy

In cancer diagnostics, traditional methods such as microscopy often depend on expert interpretation, which can be subjective and prone to variability [95]. AI enhances diagnostic accuracy and efficiency by automating image analysis, such as using augmented reality microscopes integrated with real-time AI [96]. Machine learning (ML) utilizes both supervised (labeled) and unsupervised (unlabeled) algorithms to identify diagnostic and therapeutic patterns. Additionally, ML is increasingly used in drug discovery, accelerating the identification of potential therapeutics and reducing costs by enabling predictive modeling and drug repurposing strategies. ML algorithms can analyze vast datasets from platforms like DrugBank, which provides comprehensive molecular drug data, to identify novel drug–target interactions, predict drug efficacy, and optimize lead compounds for further investigation. Predictive models, often referred to as “drug scoring,” allow for preemptive (a priori) or retrospective (a posteriori) assessments of treatment efficacy. Despite its potential, challenges remain, such as data privacy concerns, ensuring equitable access to AI technologies and ensuring algorithm reliability across diverse populations [97].

### Deep learning

Deep learning (DL), a more advanced form of ML, processes raw, unstructured data to reduce subjectivity in analysis. By modeling complex relationships, such as gene expression patterns and GBM survival, DL identifies biologically significant features that may be missed by traditional methods. DL techniques have successfully classified GBM into subtypes linked to survival outcomes, identified key progression-related genes, and uncovered hidden cellular pathways that influence disease progression. This advancement takes GBM diagnosis from traditional histological analysis to molecular profiling, leveraging modern, personalized algorithms that continuously self-learn and improve. This progression, facilitated by next-generation sequencing, holds promise for improving patient outcomes and advancing precision care [98].

## Repurposing drugs for glioblastoma treatment

Drug repurposing is a strategy that involves finding new therapeutic uses for existing FDA-approved or investigational drugs, beyond their original indications. This approach often arises from discovering off-target effects or newly identified on-target actions. In recent years, computational tools, bioinformatics, and data mining have significantly advanced drug repurposing, particularly in areas such as oncology, rare diseases, and infectious diseases. The major advantage of drug repurposing is that it allows the use of drugs with already known safety and pharmacokinetic profiles, potentially accelerating the drug development process and reducing the time and costs associated with bringing a new treatment to market.

Several notable successes in drug repurposing include aspirin, initially used for pain and inflammation, which was later repurposed for cardiovascular diseases due to its antiplatelet effects [99]; thalidomide, originally developed as a sedative and treatment for morning sickness, was withdrawn after its teratogenic effects were discovered, but was later repurposed for multiple myeloma based on its immunomodulatory properties [100]; and sildenafil, initially developed for treating angina, was repurposed for erectile dysfunction and later for pulmonary hypertension [101].

In the context of GBM, drug repurposing holds significant promise for addressing the challenges posed by tumor recurrence, resistance mechanisms, and the BBB, which hampers the efficacy of conventional treatments. Glioma stem cells (GSCs), which contribute to tumor recurrence and resistance, are another key challenge in GBM therapy. Repurposed drugs may be particularly effective in targeting these challenges due to their ability to cross the BBB and affect the biology of GBM. For example, fluoxetine, an antidepressant, has shown potential in treating GBM by crossing the BBB and directly targeting tumor cells [102]. Similarly, ibrutinib, a kinase inhibitor originally approved for B-cell malignancies, has demonstrated effectiveness in targeting GSCs. It inhibits Bruton's tyrosine kinase (BTK) and blocks the BMX-STAT3 signaling pathway, essential for GSC survival, while sparing normal progenitor cells. Clinical trials are ongoing to assess the safety and efficacy of ibrutinib in combination with standard therapies [103].

The potential for drug repurposing isn't limited to GBM alone. In the field of neurodegenerative diseases, several drugs have shown promise in early trials, offering insights for GBM treatment. For example, metformin, an antidiabetic drug, has been explored for Alzheimer’s disease (AD) due to its potential to reduce amyloid-β plaque formation [104]. Montelukast, a leukotriene receptor antagonist, has shown potential in reducing neuroinflammation in AD, although it remains in preclinical stages [105]. Exenatide, a GLP-1 receptor agonist, has demonstrated neuroprotective effects in early Parkinson's disease (PD) trials [106].

Repurposed drugs are also explored for conditions like Huntington’s disease (HD) and epilepsy. For example, tetrabenazine and deutetrabenazine have shown effectiveness in managing HD symptoms with fewer side effects [107]. Dimethyl fumarate (DMF), initially developed for multiple sclerosis, has shown promise in reducing neuronal cell death and seizures in epilepsy [108]. Ceftriaxone, a beta-lactam antibiotic, has demonstrated the ability to cross the BBB and improve survival in spinal muscular atrophy by enhancing neuroprotective protein expression [109, 110].

Table 1 provides a summary of key repurposed drugs, their original indications, and their potential applications in GBM and neurological disorders. These drugs target diverse mechanisms, including tumor suppression, neuroinflammation reduction, amyloid-β clearance, and neuronal protection. Investigating these repurposed therapies offers ^*^a promising avenue for improving patient outcomes in both oncology and neurology.

**Table 1:** Repurposed drugs and their potential applications in glioblastoma (GBM) and neurological disorders

Drug	Original indication	Repurposed application
Fluoxetine	Antidepressant	GBM: crosses BBB and targets tumor cells
Ibrutinib	B-cell malignancies (kinase inhibitor)	GBM: targets GSCs, inhibits BTK and BMX-STAT3 pathway
Metformin	Type 2 diabetes	Neurodegenerative diseases: potential to reduce amyloid-β plaque formation
Montelukast	Asthma/allergies (leukotriene receptor antagonist)	Neurodegenerative diseases: reduces neuroinflammation
Exenatide	Type 2 diabetes (GLP-1 receptor agonist)	Neurodegenerative diseases: neuroprotective effects in PD
Simvastatin	Hyperlipidemia	Neurodegenerative diseases: delays PD progression
Tetrabenazine/deutetrabenazine	Huntington’s disease (symptom management)	HD: effective in symptom management with fewer side effects
Dimethyl fumarate (DMF)	Multiple sclerosis	Epilepsy: reduces neuronal cell death and seizures
Nifedipine	Hypertension (calcium channel blocker)	Epilepsy: adjunct treatment for drug-resistant epilepsy
Ceftriaxone	Antibiotic (beta-lactam)	Neuroprotection: enhances survival of motor neuron protein in SMA

*BBB* blood–brain barrier, *BMX* bone marrow X kinase, *BTK* Bruton’s tyrosine kinase, *DMF* dimethyl fumarate, *GBM* glioblastoma, *GSCs* glioblastoma stem cells, *HD* Huntington’s disease, *PD* Parkinson’s disease, *SMA* spinal muscular atrophy, *SMN* survival of motor neuron protein, *STAT3* signal transducer and activator of transcription 3

In GBM, data-driven approaches and computational drug screening are further accelerating the identification of promising repurposed drugs. These techniques enable rapid screening of FDA-approved drugs and the discovery of potential new combination therapies tailored to the specific genetic and phenotypic profile of individual GBM patients. This approach offers a more personalized, efficient, and cost-effective strategy for treatment, with the potential to bring new hope for patients with this aggressive and difficult-to-treat cancer.

### Drug repurposing candidates

#### Typical antipsychotics (APs)

Antipsychotic drugs (Aps), such as haloperidol, trifluoperazine, fluphenazine, thioridazine, perphenazine, and chlorpromazine, are primarily used to treat psychosis, schizophrenia, bipolar disorder, and Tourette syndrome. All typical APs antagonize dopamine D2 receptors, which are present in GBM cells and involved in mitogenic signaling [111].

##### Trifluoperazine

Trifluoperazine, a typical AP, exhibits cytostatic effects on GBM cells by interacting with calmodulin subtype 2 (CaM2), leading to the release of intracellular Ca^2^⁺ ions. Dysregulation of calcium signaling induces cell death in GBM cells through the IP3R pathway, which controls calcium concentration [112].

##### Olanzapine

Olanzapine, an atypical antipsychotic, is used to treat schizophrenia, bipolar disorder, and neurological conditions like Huntington’s disease. It demonstrates anti-proliferative effects in GBM [113]. Olanzapine works by blocking serotonin and dopamine D2 receptors, inhibiting GBM cell division and movement. It may also suppress the Wnt/β-catenin signaling pathway, which is crucial for cell growth and differentiation, leading to reduced GBM cell numbers and colony formation. Additionally, olanzapine affects phospholipase D and extracellular factors, although the exact mechanisms remain unclear. The cytotoxicity of olanzapine, along with its ability to induce apoptosis and necrosis in GBM cells, likely results from the combined effects of these pathways [114].

Antipsychotic drugs have been explored for their potential anticancer effects in GBM, with studies suggesting mechanisms such as induction of apoptosis, inhibition of dopamine receptor signaling, and disruption of tumor metabolism. However, it remains uncertain whether the concentrations required for effective anticancer activity can be achieved in situ. Given this limitation, dose escalation strategies may be necessary, which could lead to an increased risk of serious side effects. Further research is needed to evaluate the therapeutic window, pharmacokinetics, and safety profile of these drugs in the context of GBM treatment.

#### Antidepressant drugs

Many antidepressant drugs are selective serotonin reuptake inhibitors (SSRIs). SSRIs increase serotonin levels at synapses, thereby activating postsynaptic neurons. SSRIs have gained attention for their potential anti-GBM properties due to their ability to cross BBB and their favorable safety profile [115].

##### Amitriptyline

Amitriptyline, a tricyclic antidepressant, works by inhibiting the reuptake of norepinephrine and serotonin [116], reducing GBM cell proliferation and stem cell properties. When combined with imipramine, it diminishes the “stemness” of GSCs, particularly under hypoxic tumor conditions. Amitriptyline decreases the expression of key stemness genes, such as Sox1, Sox2, Nestin, Ki67, and CD44, which are essential for GSC self-renewal and differentiation [117].

##### Fluvoxamine

Fluvoxamine, an SSRI, has demonstrated effectiveness in GBM treatment, with fewer peripheral side effects, making it suitable for depressed GBM patients. It inhibits GBM cell proliferation and induces apoptosis. Fluvoxamine also limits cell invasion by affecting actin polymerization and reducing the phosphorylation of key proteins like focal adhesion kinase, Akt, and mTOR. In mice models, tumor mass and invasiveness decreased, although further dose optimization is required for clinical use [118].

##### Fluoxetine

Fluoxetine induces glioma cell death without harming healthy cells. It promotes mitochondrial damage by increasing calcium influx via GluR1, leading to apoptosis through caspase activation [102]. Preclinical studies show that fluoxetine effectively reduces GBM growth, comparable to TMZ, and enhances the effects of imatinib when combined with sertraline and perphenazine [119]. These findings suggest its potential for combination therapies in GBM treatment.

#### Sodium valproate (VPA)

Sodium valproate (VPA), an antiepileptic drug, is used to treat seizures in 22–60% of GBM patients [120]. VPA is also a HDAC inhibitor, impacting tumorigenesis by promoting histone hyperacetylation and enhancing radiosensitivity [121]. VPA is frequently used in combination with other chemotherapies like TMZ in preclinical and clinical settings, particularly in TMZ-resistant tumors, where it downregulates MGMT expression. A promising Phase II trial showed improved outcomes in newly diagnosed GBM patients treated with this combination. However, VPA can inhibit various enzymes (e.g., CYP2C coenzymes, epoxide hydroxylase, UDP-glucuronosyltransferase), potentially contributing to adverse effects. Further research, including preclinical studies on diffuse intrinsic pontine glioma (DIPG), is exploring the efficacy and toxicity of VPA [122].

#### Disulfiram

Disulfiram, originally used to treat alcoholism, shows promise as a repurposed therapy for GBM [123]. It inhibits ALDH, leading to acetaldehyde buildup and alcohol intolerance. Preclinical studies suggest that disulfiram inhibits GBM cell proliferation and self-renewal, particularly in TMZ-resistant cells, while sparing normal cells [124]. It also crosses the BBB [123] and decreases MGMT expression, enhancing its cytotoxic effects. Disulfiram’s anti-tumor activity involves targeting ALDH, disrupting the proteasome and NF-κB pathways, and affecting the p97 pathway. Its conversion to diethyldithiocarbamate, a copper-chelating agent, increases cytotoxicity when combined with copper [125]. Despite its efficacy, careful dosing is essential to avoid copper toxicity. Given its safety profile and BBB penetration, disulfiram is a promising candidate for GBM and potentially pediatric brain tumor treatment, though further research is required.

#### Mebendazole

Mebendazole, an FDA-approved anthelmintic drug with microtubule-inhibiting properties, holds promise for GBM treatment. Its effectiveness in crossing the BBB depends on its crystalline polymorph form, with polymorphs B and C being more effective for CNS delivery. Mebendazole is generally safe, although high concentrations may cause bone marrow and liver toxicity. It is thought to inhibit protein kinase activity, and further investigation is needed to understand its role in cell death and microtubule destabilization [126]. Preclinical studies have shown that polymorph C improves survival in mice with GL261 gliomas, particularly by affecting tumor invasiveness [126]. Given these promising results, mebendazole is considered a potential substitute for vincristine in neuro-oncology for GBM treatment.

#### Clomiphene

Clomiphene, a selective estrogen receptor modulator used for female infertility and hypogonadism, shows potential as a repurposed drug for GBM. It works by antagonizing estrogen receptors in the hypothalamus, leading to the release of luteinizing hormone and follicle-stimulating hormone. Recent studies have shown that clomiphene inhibits mutant IDH1, an enzyme involved in tumorigenesis, preventing the accumulation of D-2-hydroxyglutarate, which promotes carcinogenesis by inhibiting histone demethylase. In vitro and in vivo studies demonstrate that clomiphene induces apoptosis in IDH1-mutant glioma cells with minimal liver and kidney toxicity. Furthermore, it reduces IDH1-promoted H3K9me3 levels in mouse xenografts, suggesting potential therapeutic benefits [127]. Further research is needed to explore clomiphene’s role as a targeted treatment for GBM.

#### Metformin

Metformin, an oral antidiabetic drug, is explored as a repurposed treatment for GBM. It reduces gluconeogenesis, increases glycolysis, and improves insulin sensitivity, with observed potential in cancer prevention. Metformin has been shown to exhibit anti-gliomagenic potential by suppressing GBM cell proliferation and migration, inducing apoptosis, reversing TMZ-resistance, suppressing self-renewal, and inhibiting GSC stemness. In xenograft models, metformin has been shown to regress tumors and increase survival. It is believed that metformin exerts its anti-tumor effects through multiple mechanisms, including acting on complex I of the mitochondrial electron transport chain. This leads to an increase in AMP, which upregulates LKB1-AMP-activated protein kinase, inhibiting the mammalian mTORC1. When mTORC1 is low, cancer cell proliferation is also reduced, and the switch from mitochondrial ATP production to glycolytic ATP production occurs, resulting in increased lactate production. Metformin also suppresses the AMPK and STAT3 pathways [128–131].

Notably, metformin affects miRNAs that regulate post-translational gene expression, influencing energy metabolism, cell division, and stem cells. For example, phenformin, a lipophilic analog of metformin, upregulates miR-124 and let-7, which are crucial for GSC self-renewal. Biguanides improve the bioavailability of let-7 by downregulating H19, the binding partner of let-7, promoting let-7 suppression of the oncogene HMGA2 [132]. Additionally, metformin inhibits glutamate dehydrogenase and decreases glutaminolysis and oncometabolite D-2-HG in IDH1/2-mutated gliomas [128]. Metformin can selectively target chloride intracellular channel-1 in GBM, resulting in G1 arrest of GSCs [133, 134].

Given its multiple mechanisms of action, metformin is well-suited for repositioning as a treatment for heterogeneous tumors like GBM. When administered in combination with chloroquine, it has been shown to reduce the size of IDH1-mutated glioma tumors in clinical trials. In vivo experiments show that both metformin and chloroquine effectively penetrate the BBB. However, new data suggest that gliomas may overexpress metformin efflux transporters, raising concerns about whether sufficient intratumoral concentrations can be achieved [128].

#### Statins

Statins, widely used lipid-lowering drugs, show potential in treating GBM by inhibiting the HMG-CoA to mevalonate conversion, decreasing isoprenoids synthesis and disrupting prenylation of GTP-binding proteins, such as Ras, Rac, and Rho involved in cancer proliferation. Epidemiological studies on pre-operative statin use in GBM patients yielded inconclusive survival outcomes. However, in vitro studies reveal time- and dose-dependent cytotoxic effects of statins on GBM cells [135]. Statins influence multiple pathways, including TNF-related apoptosis-inducing ligand (TRAIL) protein upregulation, increased pro-apoptotic protein Bim levels, MAPK pathway downregulation, ERK1/2 and Ras/PI3K/Akt inhibition, and c-Jun N-terminal kinase (JNK) activation, leading to tumor cell death [135]. Furthermore, statins modulate inflammation via NLRP3 inflammasomes and NF-κB pathways. Although clinical trials have shown limited success, statins are being investigated in combination therapies. For example, lovastatin combined with TMZ and sirolimus enhances chemotherapy effects, while pairing statins with cyclooxygenase-2 (COX-2) inhibitors shows promise in synergistically inhibiting tumor growth [135, 136]. Among repurposed drug candidates, statins appear to be a safer class for dose escalation, given their long-standing use in cardiovascular disease and their relatively well-characterized safety profiles. Notably, an ongoing Phase 0/1 clinical trial (ClinicalTrials.gov: NCT05977738) is currently evaluating the safety and tumor drug concentrations of pitavastatin in GBM patients, providing key insights into the feasibility of statin-based therapies for GBM treatment.

A growing emphasis is being placed on Phase 0/Window of Opportunity trials to assess the pharmacokinetics (PK) and pharmacodynamics (PD) of investigational therapies in GBM. Given the limitations imposed by the BBB and the molecular heterogeneity of GBM, there is a shifting consensus that drugs should not be administered to GBM patients without first confirming their ability to reach and effectively modify their intended target. Phase 0 trials provide early insights into drug distribution, target engagement, and biological response, allowing for data-driven decisions on whether to advance or discontinue a drug candidate. This approach not only enhances trial efficiency and patient safety but also accelerates the development of more effective, targeted therapies for GBM.

Table 2 summarizes key repurposed non-oncological drugs, their original indications, and potential GBM applications, targeting tumor progression, chemoresistance, and immune modulation. Further research is needed to assess their clinical efficacy, optimal dosing, and BBB penetration. Table 3 outlines key therapeutic targets, their mechanisms, and clinical implications in GBM treatment. Current therapies, such as bevacizumab and IDH inhibitors, show promise, while others like EGFR inhibitors and immune checkpoint therapies have yielded mixed results.

**Table 2:** Repurposed non-oncological drugs and their potential applications in glioblastoma (GBM) treatment

Drug	Original indication	Repurposed GBM application
Trifluoperazine	Typical antipsychotic for psychosis, schizophrenia	Induces cell death via calcium signaling dysregulation
Olanzapine	Atypical antipsychotic for schizophrenia, bipolar disorder	Blocks serotonin and dopamine receptors, suppresses Wnt/β-catenin signaling
Amitriptyline	Tricyclic antidepressant	Reduces GBM cell proliferation and stem cell properties
Fluvoxamine	Selective serotonin reuptake inhibitor (SSRI)	Inhibits proliferation, induces apoptosis, limits invasion
Sodium valproate (VPA)	Antiepileptic drug for seizures	Enhances radiosensitivity, downregulates MGMT expression
Disulfiram	Alcoholism treatment	Targets ALDH, proteasome and NF-ĸB pathways, enhances cytotoxicity with copper
Mebendazole	Anthelmintic drug	Affects microtubule destabilization, reduces tumor invasiveness
Clomiphene	Selective estrogen receptor modulator for infertility	Inhibits mutant IDH1, induces apoptosis in IDH1-mutant glioma cells
Metformin	Anti-diabetic drug	Suppresses proliferation, induces apoptosis, reverses TMZ-resistance
Statins	Lipid-lowering drug	Cytotoxic effects on GBM cells, modulate inflammation, enhance combination therapies

*5-HT* 5-hydroxytryptamine (serotonin), *ALDH* aldehyde dehydrogenase, *DA* dopamine, *GBM* glioblastoma, *IDH1* isocitrate dehydrogenase 1, *MGMT* O6 methylguanine-DNA methyltransferase, *NF-κB* nuclear factor kappa B, *SERM* selective estrogen receptor modulator, *SSRI* selective serotonin reuptake inhibitor, *TCA* tricyclic antidepressant, *TMZ* temozolomide, *VPA* sodium valproate

**Table 3:** Summary of key therapeutic targets and molecular pathways in glioblastoma (GBM) treatment

Therapeutic target	Mechanism of action	Molecular pathways involved	Clinical implications in GBM
EGFR	Inhibitors block receptor signaling to prevent tumor growth and proliferation	EGFR/RTK/PI3K/AKT	EGFR inhibitors like erlotinib and gefitinib have shown mixed efficacy in clinical trials
PD-1/PD-L1	Immune checkpoint inhibitors restore T-cell activation and enhance immune response	Immune checkpoint pathway	PD-1/PD-L1 inhibitors (nivolumab, pembrolizumab) are under evaluation but show limited response in GBM
VEGF	Anti-angiogenic agents reduce tumor vascularization, limiting nutrient supply	VEGF/VEGFR signaling	Bevacizumab is FDA approved for recurrent GBM but does not significantly improve survival
IDH1/2	IDH inhibitors reduce oncometabolite production and promote differentiation	Metabolic pathway (TCA cycle)	IDH inhibitors (ivosidenib, vorasidenib) show promise in IDH-mutant gliomas
MGMT	MGMT inhibitors prevent DNA repair in tumor cells, enhancing chemotherapy efficacy	DNA repair pathway	MGMT methylation status is a key predictor of temozolomide response
PI3K/AKT/mTOR Pathway	Pathway inhibitors block cell survival and proliferation signaling	PI3K/AKT/mTOR signaling	Targeted inhibitors (e.g., Rapamycin) show potential in overcoming therapy resistance
Wnt/β-catenin pathway	Wnt pathway inhibitors disrupt tumor cell self-renewal and invasion	Wnt/Î^2^-catenin pathway	Blocking Wnt/β-catenin could reduce GBM invasion and self-renewal properties
Bcl-2	Bcl-2 inhibitors induce apoptosis in GBM cells	Apoptosis regulation	Bcl-2 inhibition may enhance apoptosis in GBM, though resistance mechanisms exist
HDACs	HDAC inhibitors promote tumor suppressor gene activation and reduce GBM progression	Epigenetic regulation	HDAC inhibitors (vorinostat, valproic acid) are under clinical investigation for GBM treatment
Glutathione pathway	Targeting glutathione reduces oxidative stress resistance in GBM cells	Oxidative stress pathway	Reducing glutathione in GBM cells may enhance sensitivity to oxidative stress-based therapies

*GBM* Glioblastoma, *EGFR* epidermal growth factor receptor, *PD-1* programmed death receptor 1, *PD-L1* programmed death-ligand 1, *VEGF* vascular endothelial growth factor, *VEGFR* vascular endothelial growth factor receptor, *IDH1/2* isocitrate dehydrogenase 1/2, *MGMT* O6-methylguanine-DNA methyltransferase, *PI3K* phosphoinositide 3-kinase, *AKT* protein kinase B, *mTOR* mammalian target of rapamycin, *RTK* receptor tyrosine kinase, *TCA Cycle* tricarboxylic acid cycle (Krebs cycle), *Wnt/β-catenin* wingless-related integration site/beta-catenin, *Bcl-2* B-cell lymphoma 2, *HDACs* histone deacetylases

## Research gaps and future prospects

Despite significant advancements in GBM treatment, therapy resistance, the TME, and drug delivery challenges continue to hinder effective treatment. Addressing these key areas is essential to improving long-term patient outcomes.

### Mechanisms of resistance

Resistance to GBM therapies, particularly TMZ, remains a major obstacle. MGMT overexpression counteracts TMZ efficacy by repairing DNA damage, suggesting that MGMT inhibitors could help restore drug sensitivity. P-glycoprotein (P-gp), a membrane transporter, actively expels chemotherapeutic agents, reducing drug effectiveness; thus, targeting P-gp inhibitors could improve chemotherapy uptake. Additionally, GBM stem cells (GSCs) contribute to tumor recurrence and therapy resistance. Investigating key molecular pathways, such as Wnt/β-catenin and Notch signaling, may provide novel strategies to target GSCs and enhance GBM treatment.

### Tumor microenvironment and immune evasion

The GBM TME is highly immunosuppressive, facilitating immune evasion and therapy resistance. PD-1/PD-L1 and immunosuppressive cytokines (e.g., TGF-β) play critical roles in this process, making immune checkpoint inhibitors a promising avenue to restore immune surveillance. Additionally, hypoxia and exosome secretion contribute to tumor progression and resistance by promoting intercellular communication and metastasis. Strategies such as hypoxia-targeted therapies and exosome inhibition could enhance current immunotherapies.

### Technological advancements in GBM monitoring

Recent innovations have improved GBM diagnosis, treatment monitoring, and personalized care. Circulating tumor DNA (ctDNA) liquid biopsies enable real-time tumor monitoring, mutation detection, and treatment response assessment. Extracellular vesicles (EVs), which influence GBM progression, are also emerging as potential biomarkers. Furthermore, single-cell RNA sequencing (scRNA-seq) helps characterize tumor heterogeneity, facilitating targeted therapy development. Artificial intelligence (AI)-driven multi-omics analysis offers new opportunities for optimizing diagnostics, predicting therapy responses, and identifying novel drug targets.

### Emerging therapies and immunotherapy

Immunotherapy remains a promising avenue for GBM treatment. Chimeric antigen receptor (CAR) T-cell therapy, which engineers T-cells to target tumor-specific antigens (e.g., EGFRvIII), is under clinical investigation. However, overcoming GBM’s immunosuppressive TME remains a challenge. Oncolytic viruses, genetically engineered to selectively infect and kill tumor cells, are another emerging approach, particularly in combination with immune checkpoint inhibitors. While immune-based therapies have transformed treatment in other cancers, their application in GBM requires further refinement to enhance efficacy.

### Epigenetic modulation and combination therapies

Epigenetic alterations play a crucial role in GBM progression and therapy resistance. Histone deacetylase inhibitors (HDACis) have shown potential in resensitizing GBM cells to chemotherapy and promoting apoptosis. Combining epigenetic therapies with TMZ or radiation could enhance treatment effectiveness. Additionally, combination therapies integrating chemotherapy, targeted agents, and immunotherapies may address resistance mechanisms and improve survival outcomes.

### Drug delivery and blood–brain barrier (BBB) penetration

The BBB remains a major challenge in GBM treatment, limiting drug penetration into the tumor. Nanocarriers, including liposomes and nanoparticles, offer promising strategies for targeted drug delivery, bypassing the BBB and improving therapeutic specificity. Temporary BBB disruption techniques allow drug passage but require careful modulation to avoid damage to healthy brain tissue. Advancements in nanoparticle engineering to target GBM-specific biomarkers could enhance drug delivery precision while minimizing systemic toxicity.

### Personalized approaches and clinical trials

Personalized medicine is transforming GBM treatment by tailoring therapies to individual tumor characteristics. Biomarkers such as MGMT methylation and EGFR variants guide treatment selection, optimizing patient outcomes. The integration of multi-omics data (genomic, proteomic, and transcriptomic) further enhances precision therapy design. Clinical trials exploring drug repurposing, combination therapies, and adaptive trial designs are crucial for translating promising preclinical findings into clinical applications.

As research and technology continue to evolve, a multidisciplinary approach combining molecular insights, technological innovations, and novel therapies will be essential for advancing GBM treatment and improving patient survival.

## Summary

GBM remains one of the most aggressive and lethal brain tumors, characterized by significant heterogeneity, therapy resistance, and the challenge of overcoming the blood–brain BBB. Standard treatments, including surgery, radiotherapy, and temozolomide, provide limited survival benefits, largely due to therapy evasion mechanisms such as GBM stem cell survival, P-glycoprotein activity, and MGMT overexpression, leading to recurrence and resistance.

Emerging therapeutic strategies such as targeted therapies, immunotherapy, tumor-treating fields, and drug repurposing (e.g., fluoxetine, metformin, and disulfiram) offer new hope in improving treatment efficacy. Additionally, advanced diagnostics like liquid biopsy, next-generation sequencing, and AI-driven insights are paving the way for personalized and adaptive treatment approaches.

However, significant research gaps remain in targeting GSCs, overcoming the BBB, and modulating the TME. Addressing these challenges is crucial to improving long-term outcomes. Future directions in GBM research include personalized medicine, which tailors treatments based on patient-specific molecular profiles, and combination therapies that synergize existing and novel agents. Advancements in nanotechnology and innovative drug delivery methods hold promise in enhancing therapeutic penetration across the BBB. Moreover, the integration of AI and machine learning in drug discovery and personalized treatment planning will refine therapeutic decision-making and enable real-time tumor monitoring.

While the road ahead remains challenging, multidisciplinary collaboration, technological innovations, and clinical trials will be essential in translating research into tangible improvements for patient care. With continued progress, the future of GBM treatment is shifting toward more personalized, targeted, and effective therapeutic strategies, offering renewed hope for patients and their families.

## Data Availability

No datasets were generated or analyzed during the current study.

## Glossary

AI (Artificial Intelligence): A field of computer science that enables machines to perform tasks that typically require human intelligence, such as learning, problem-solving, and decision-making.
ALDH (Aldehyde Dehydrogenase): An enzyme that helps metabolize aldehydes, playing a role in detoxification and cellular regulation.
BTK (Bruton’s Tyrosine Kinase): An enzyme involved in the signaling pathways of B-cells, playing a role in immune cell function and implicated in certain cancers.
CAR-T (Chimeric Antigen Receptor T-Cell Therapy): A type of immunotherapy that modifies T-cells to express receptors specific to cancer cells, aiding the immune system in targeting and killing those cells.
COX-2 (Cyclooxygenase-2): An enzyme that promotes inflammation and is associated with the development of various cancers and other diseases.
ctDNA (Circulating Tumor DNA): Fragments of DNA shed by tumors into the bloodstream, used in liquid biopsy tests to detect and monitor cancer.
CTLA-4 (Cytotoxic T-Lymphocyte-Associated Protein 4): A protein on immune cells that regulates immune responses and can be targeted in cancer immunotherapy to enhance immune activity.
DL (Deep Learning): A subset of machine learning that uses neural networks with many layers to analyze complex patterns in data.
EGFR (Epidermal Growth Factor Receptor): A protein on cell surfaces involved in cell growth and differentiation, often mutated in various cancers.
EVs (Extracellular Vesicles): Small membrane-bound particles secreted by cells that contain proteins, lipids, and genetic material, involved in intercellular communication.
GBM (Glioblastoma): An aggressive, fast-growing form of brain cancer with a poor prognosis.
GSCs (Glioblastoma Stem Cells): Cancer stem cells found in GBM that are thought to contribute to tumor growth and resistance to treatment.
HDACi (Histone Deacetylase Inhibitor): A class of drugs that block enzymes involved in modifying the structure of chromatin, influencing gene expression and cancer treatment.
HIF (Hypoxia-Inducible Factor): A transcription factor that regulates cellular responses to low oxygen conditions, often upregulated in tumors to aid their survival.
IDH (Isocitrate Dehydrogenase): An enzyme involved in metabolism, mutations of which are linked to certain cancers, including gliomas.
MDSCs (Myeloid-Derived Suppressor Cells): Immune cells that suppress immune responses, often exploited by tumors to evade detection by the immune system.
MGMT (O6-Methylguanine-DNA Methyltransferase): An enzyme that repairs DNA damage, often associated with resistance to chemotherapy in cancer.
ML (Machine Learning): A branch of AI that uses algorithms to analyze data, learn from it, and make predictions or decisions based on that data.
MMPs (Matrix Metalloproteinases): A group of enzymes that break down the extracellular matrix, playing a role in tissue remodeling and cancer metastasis.
NGS (Next-Generation Sequencing): A high-throughput DNA sequencing technology that enables rapid sequencing of large volumes of genetic material, often used in cancer research.
NK (Natural Killer): A type of immune cell that plays a critical role in the body’s defense against tumors and infections by killing abnormal cells.
PD-1 (Programmed Death-1): A protein found on immune cells that helps regulate the immune response; targeting PD-1 in cancer therapy can enhance immune system activity against tumors.
PTEN (Phosphatase and Tensin Homolog): A tumor suppressor gene involved in cell cycle regulation, often mutated in various cancers.
RB1 (Retinoblastoma Gene 1): A gene that regulates the cell cycle and suppresses tumor formation, frequently mutated in cancers like retinoblastoma and others.
scRNA-seq (Single-Cell RNA Sequencing): A technique for analyzing the gene expression profiles of individual cells, providing insights into cellular diversity and function within tissues.
SSRIs (Selective Serotonin Reuptake Inhibitors): A class of drugs commonly used to treat depression and anxiety by increasing serotonin levels in the brain.
TME (Tumor Microenvironment): The surrounding cellular environment of a tumor, including immune cells, blood vessels, and other factors, that influence cancer progression and treatment response.
TMZ (Temozolomide): A chemotherapy drug commonly used to treat GBM and other cancers, functioning by damaging DNA and inhibiting cancer cell growth.
TP53 (Tumor Protein p53): A tumor suppressor protein that regulates the cell cycle and prevents cancer formation; mutations in TP53 are common in many cancers.
VEGF (Vascular Endothelial Growth Factor): A protein that stimulates the formation of blood vessels, often targeted in cancer therapy to prevent tumor growth.

## References

[1] Louis DN, Perry A, Wesseling P, Brat DJ, Cree IA, Figarella-Branger D, et al. The 2021 WHO classification of tumors of the central nervous system: a summary. Neuro Oncol. 2021;23(8):1231–51. 34185076 10.1093/neuonc/noab106 PMC8328013

[2] Louis DN, Perry A, Reifenberger G, von Deimling A, Figarella-Branger D, Cavenee WK, et al. The 2016 world health organization classification of tumors of the central nervous system: a summary. Acta Neuropathol. 2016;131(6):803–20. 27157931 10.1007/s00401-016-1545-1

[3] McKinnon C, Nandhabalan M, Murray SA, Plaha P. Glioblastoma: clinical presentation, diagnosis, and management. BMJ. 2021;374:574012. 10.1136/bmj.n1560 34261630

[4] Oronsky B, Reid TR, Oronsky A, Sandhu N, Knox SJ. A review of newly diagnosed glioblastoma. Front Oncol. 2021;10:574012. 33614476 10.3389/fonc.2020.574012 PMC7892469

[5] Ntafoulis I, Koolen SL, van Tellingen O, den Hollander CW, Sabel-Goedknegt H, Dijkhuizen S, et al. A repurposed drug selection pipeline to identify CNS-penetrant drug candidates for glioblastoma. Pharmaceuticals. 2024;17(12):1687. 39770529 10.3390/ph17121687 PMC11678797

[6] Lee JH, Lee JE, Kahng JY, Kim SH, Park JS, Yoon SJ, et al. Human glioblastoma arises from subventricular zone cells with low-level driver mutations. Nature. 2018;560(7717):243–7. 30069053 10.1038/s41586-018-0389-3

[7] Stoyanov GS, Dzhenkov DL. On the concepts and history of glioblastoma multiforme—morphology. Genet Epigenet Folia Med (Plovdiv). 2018;60(1):48–66. 10.1515/folmed-2017-0069 29668458

[8] Brennan CW, Verhaak RGW, McKenna A, Campos B, Noushmehr H, Salama SR, et al. The somatic genomic landscape of glioblastoma. Cell. 2013;155(2):462–77. 24120142 10.1016/j.cell.2013.09.034 PMC3910500

[9] Kane JR. The role of brain vasculature in glioblastoma. Mol Neurobiol. 2019;56(9):6645–53. 30911935 10.1007/s12035-019-1561-y

[10] Watson DC, Bayik D, Storevik S, Moreino SS, Sprowls SA, Han J, et al. GAP43-dependent mitochondria transfer from astrocytes enhances glioblastoma tumorigenicity. Nat Cancer. 2023;4(5):648–64. 37169842 10.1038/s43018-023-00556-5 PMC10212766

[11] Krishna S, Choudhury A, Keough MB, Seo K, Ni L, Kakaizada S, et al. Glioblastoma remodelling of human neural circuits decreases survival. Nature. 2023;617(7961):599–607. 37138086 10.1038/s41586-023-06036-1 PMC10191851

[12] Huang Y, Hoffman C, Rajappa P, Kim JH, Hu W, Huse J, et al. Oligodendrocyte progenitor cells promote neovascularization in glioma by disrupting the blood-brain barrier. Cancer Res. 2014;74(4):1011–21. 24371228 10.1158/0008-5472.CAN-13-1072

[13] Roesch S, Rapp C, Dettling S, Herold-Mende C. When immune cells turn bad—tumor-associated microglia/macrophages in glioma. Int J Mol Sci. 2018;19(2):436. 29389898 10.3390/ijms19020436 PMC5855658

[14] Hernández A, Domènech M, Muñoz-Mármol AM, Carrato C, Balana C. Glioblastoma: relationship between metabolism and immunosuppressive microenvironment. Cells. 2021;10(12):3529. 34944036 10.3390/cells10123529 PMC8700075

[15] Heddleston JM, Li Z, McLendon RE, Hjelmeland AB, Rich JN. The hypoxic microenvironment maintains glioblastoma stem cells and promotes reprogramming towards a cancer stem cell phenotype. Cell Cycle. 2009;8(20):3274–85. 19770585 10.4161/cc.8.20.9701 PMC2825672

[16] Vaupel P, Multhoff G. Accomplices of the hypoxic tumor microenvironment compromising antitumor immunity: adenosine, lactate, acidosis, vascular endothelial growth factor, potassium ions, and phosphatidylserine. Front Immunol. 2017. 10.3389/fimmu.2017.01887. 29312351 10.3389/fimmu.2017.01887 PMC5742577

[17] Oliver L, Lalier L, Salaud C, Heymann D, Cartron PF, Vallette FM. Drug resistance in glioblastoma: are persisters the key to therapy? Cancer Drug Resist. 2020;3(3):287. 35582442 10.20517/cdr.2020.29 PMC8992484

[18] Zhang YW, Su Y, Volpert OV, Woude GF. Hepatocyte growth factor/scatter factor mediates angiogenesis through positive VEGF and negative thrombospondin 1 regulation. Proc Natl Acad Sci USA. 2003;100(22):12718–23. 14555767 10.1073/pnas.2135113100 PMC240684

[19] Baish JW, Stylianopoulos T, Lanning RM, Kamoun WS, Fukumura D, Munn LL, et al. Scaling rules for diffusive drug delivery in tumor and normal tissues. Proc Natl Acad Sci USA. 2011;108(5):1799–803. 21224417 10.1073/pnas.1018154108 PMC3033252

[20] Lin JL, Wang MJ, Lee D, Liang CC, Lin S. Hypoxia-inducible factor-1α regulates matrix metalloproteinase-1 activity in human bone marrow-derived mesenchymal stem cells. FEBS Lett. 2008;582(17):2615–9. 18588890 10.1016/j.febslet.2008.06.033

[21] Robinson K, Tiriveedhi V. Perplexing role of P-glycoprotein in tumor microenvironment. Front Oncol. 2020. 10.3389/fonc.2020.00265. 32195185 10.3389/fonc.2020.00265 PMC7066112

[22] Da Ros M, De Gregorio V, Iorio AL, Giunti L, Guidi M, De Martino M, et al. Glioblastoma chemoresistance: the double play by microenvironment and blood-brain barrier. Int J Mol Sci. 2018;19(10):2879. 30248992 10.3390/ijms19102879 PMC6213072

[23] Bogdanov A, Bogdanov A, Chubenko V, Volkov N, Moiseenko F, Moiseyenko V. Tumor acidity: from hallmark of cancer to target of treatment. Front Oncol. 2022;12:979154. 36106097 10.3389/fonc.2022.979154 PMC9467452

[24] Jamali F, Lan K, Daniel P, Petrecca K, Sabri S, Abdulkarim B. Synergistic dual targeting of thioredoxin and glutathione systems irrespective of p53 in glioblastoma stem cells. Antioxidants. 2024;13(10):1201. 39456455 10.3390/antiox13101201 PMC11504866

[25] Posti JP, Bori M, Kauko T, Sankinen M, Nordberg J, Rahi M, et al. Presenting symptoms of glioma in adults. Acta Neurol Scand. 2015;131(2):88–93. 25263022 10.1111/ane.12285

[26] Chen H, Judkins J, Thomas C, Wu M, Khoury L, Benjamin CG, et al. Mutant IDH1 and seizures in patients with glioma. Neurology. 2017;88(19):1805–13. 28404805 10.1212/WNL.0000000000003911 PMC5419985

[27] Bijalwan G, Shrivastav AK, Mallik S, Dubey MK. Glioblastoma multiforme-a rare type of cancer: a narrative review. Cancer Res Stat Treat. 2024;7(3):340–51.

[28] Sanger F, Nicklen S, Coulson AR. DNA sequencing with chain-terminating inhibitors. Proc Natl Acad Sci USA. 1977;74(12):5463–7. 271968 10.1073/pnas.74.12.5463 PMC431765

[29] Metzker ML. Emerging technologies in DNA sequencing. Genome Res. 2005;15(12):1767–76. 16339375 10.1101/gr.3770505

[30] Hert DG, Fredlake CP, Barron AE. Advantages and limitations of next-generation sequencing technologies: a comparison of electrophoresis and non-electrophoresis methods. Electrophoresis. 2008;29(23):4618–26. 19053153 10.1002/elps.200800456

[31] Kircher M, Kelso J. High-throughput DNA sequencing–concepts and limitations. BioEssays. 2010;32(6):524–36. 20486139 10.1002/bies.200900181

[32] Gullapalli RR, Desai KV, Santana-Santos L, Kant JA, Becich MJ. Next generation sequencing in clinical medicine: challenges and lessons for pathology and biomedical informatics. J Pathol Inform. 2012;3:40. 23248761 10.4103/2153-3539.103013 PMC3519097

[33] Metzker ML. Sequencing technologies—the next generation. Nat Rev Genet. 2010;11(1):31–46. 19997069 10.1038/nrg2626

[34] Wang Y, Yang Q, Wang Z. The evolution of nanopore sequencing. Front Genet. 2014;5:449. 25610451 10.3389/fgene.2014.00449 PMC4285804

[35] Kasianowicz JJ, Brandin E, Branton D, Deamer DW. Characterization of individual polynucleotide molecules using a membrane channel. Proc Natl Acad Sci USA. 1996;93(24):13770–3. 8943010 10.1073/pnas.93.24.13770 PMC19421

[36] Branton D, Deamer DW, Marziali A, Bayley H, Benner SA, Butler T, et al. The potential and challenges of nanopore sequencing. Nat Biotechnol. 2008;26(10):1146–53. 18846088 10.1038/nbt.1495 PMC2683588

[37] Jain M, Koren S, Miga KH, Quick J, Rand AC, Sasani TA, et al. Nanopore sequencing and assembly of a human genome with ultra-long reads. Nat Biotechnol. 2018;36(4):338–45. 29431738 10.1038/nbt.4060 PMC5889714

[38] Euskirchen P, Bielle F, Labreche K, Kloosterman WP, Rosenberg S, Daniau M, et al. Same-day genomic and epigenomic diagnosis of brain tumors using real-time nanopore sequencing. Acta Neuropathol. 2017;134(5):691–703. 28638988 10.1007/s00401-017-1743-5 PMC5645447

[39] Cancer Genome Atlas Research N. Comprehensive genomic characterization defines human glioblastoma genes and core pathways. Nature. 2008;455(7216):1061–8. 18772890 10.1038/nature07385 PMC2671642

[40] Yan H, Parsons DW, Jin G, McLendon R, Rasheed BA, Yuan W, et al. IDH1 and IDH2 mutations in gliomas. N Engl J Med. 2009;360(8):765–73. 19228619 10.1056/NEJMoa0808710 PMC2820383

[41] Pang L, Hu J, Li F, Yuan H, Yan M, Liao G, et al. Discovering rare genes contributing to cancer stemness and invasive potential by GBM single-cell transcriptional analysis. Cancers. 2019;11(12):2025. 31888172 10.3390/cancers11122025 PMC6966673

[42] Meyer M, Reimand J, Lan X, Head R, Zhu X, Kushida M, et al. Single cell-derived clonal analysis of human glioblastoma links functional and genomic heterogeneity. Proc Natl Acad Sci USA. 2015;112(3):851–6. 25561528 10.1073/pnas.1320611111 PMC4311802

[43] Muller S, Liu SJ, Di Lullo E, Malatesta M, Pollen AA, Nowakowski TJ, et al. Single-cell sequencing maps gene expression to mutational phylogenies in PDGF- and EGF-driven gliomas. Mol Syst Biol. 2016;12(9):889. 27888226 10.15252/msb.20166969 PMC5147052

[44] Romani M, Pistillo MP, Banelli B. Epigenetic targeting of glioblastoma. Front Oncol. 2018;8:448. 30386738 10.3389/fonc.2018.00448 PMC6198064

[45] Li B, Carey M, Workman JL. The role of chromatin during transcription. Cell. 2007;128(4):707–19. 17320508 10.1016/j.cell.2007.01.015

[46] Marino-Ramirez L, Kann MG, Shoemaker BA, Landsman D. Histone structure and nucleosome stability. Expert Rev Proteom. 2005;2(5):719–29. 10.1586/14789450.2.5.719 PMC1831843 16209651

[47] Jenuwein T, Allis CD. Translating the histone code. Science. 2001;293(5532):1074–80. 11498575 10.1126/science.1063127

[48] Schwartzentruber J, Korshunov A, Liu XY, Jones DT, Pfaff E, Jacob K, et al. Driver mutations in histone H3.3 and chromatin remodelling genes in paediatric glioblastoma. Nature. 2012;482(7384):226–31. 22286061 10.1038/nature10833

[49] Bannister AJ, Kouzarides T. Regulation of chromatin by histone modifications. Cell Res. 2011;21(3):381–95. 21321607 10.1038/cr.2011.22 PMC3193420

[50] Sekine SI, Ehara H, Kujirai T, Kurumizaka H. Structural perspectives on transcription in chromatin. Trends Cell Biol. 2024;34(3):211–24. 37596139 10.1016/j.tcb.2023.07.011

[51] Yekta RF, Farahani M, Koushki M, Amiri-Dashatan N. Deciphering the potential role of post-translational modifications of histones in gastrointestinal cancers: a proteomics-based review with therapeutic challenges and opportunities. Front Oncol. 2024;14:1481426. 39497715 10.3389/fonc.2024.1481426 PMC11532047

[52] Choi SA, Kwak PA, Park CK, Wang KC, Phi JH, Lee JY, et al. A novel histone deacetylase inhibitor, CKD5, has potent anti-cancer effects in glioblastoma. Oncotarget. 2017;8(6):9123–33. 27852054 10.18632/oncotarget.13265 PMC5354719

[53] Ohgaki H, Kleihues P. Genetic pathways to primary and secondary glioblastoma. Am J Pathol. 2007;170(5):1445–53. 17456751 10.2353/ajpath.2007.070011 PMC1854940

[54] Dirkse A, Golebiewska A, Buder T, Nazarov PV, Muller A, Poovathingal S, et al. Stem cell-associated heterogeneity in glioblastoma results from intrinsic tumor plasticity shaped by the microenvironment. Nat Commun. 2019;10(1):1787. 30992437 10.1038/s41467-019-09853-z PMC6467886

[55] Stupp R, Taillibert S, Kanner A, Read W, Steinberg DM, Lhermitte B, et al. Effect of tumor-treating fields plus maintenance temozolomide vs maintenance temozolomide alone on survival in patients with glioblastoma. JAMA. 2017;318(23):2306–16. 29260225 10.1001/jama.2017.18718 PMC5820703

[56] An Z, Aksoy O, Zheng T, Fan QW, Weiss WA. Epidermal growth factor receptor and EGFRvIII in glioblastoma: signaling pathways and targeted therapies. Oncogene. 2018;37(12):1561–75. 29321659 10.1038/s41388-017-0045-7 PMC5860944

[57] Mellinghoff IK, Ellingson BM, Touat M, Maher E, De La Fuente MI, Holdhoff M, et al. Ivosidenib in isocitrate dehydrogenase 1-mutated advanced glioma. J Clin Oncol. 2020;38(29):3398–406. 32530764 10.1200/JCO.19.03327 PMC7527160

[58] Ferrara N, Adamis AP. Ten years of anti-vascular endothelial growth factor therapy. Nat Rev Drug Discov. 2016;15(6):385–403. 26775688 10.1038/nrd.2015.17

[59] Frederico SC, Hancock JC, Brettschneider EES, Ratnam NM, Gilbert MR, Terabe M. Making a cold tumor hot: the role of vaccines in the treatment of glioblastoma. Front Oncol. 2021;11:672508. 34041034 10.3389/fonc.2021.672508 PMC8141615

[60] Zhao T, Li C, Ge H, Lin Y, Kang D. Glioblastoma vaccine tumor therapy research progress. Chin Neurosurg J. 2022;8(1):2. 35045874 10.1186/s41016-021-00269-7 PMC8766628

[61] Wu W, Wu Y, Mayer K, von Rosenstiel C, Schecker J, Baur S, et al. Lipid peroxidation plays an important role in chemotherapeutic effects of temozolomide and the development of therapy resistance in human glioblastoma. Transl Oncol. 2020;13(3):100748. 32087559 10.1016/j.tranon.2020.100748 PMC7033364

[62] Sheykhzadeh S, Luo M, Peng B, White J, Abdalla Y, Tang T, et al. Transferrin-targeted porous silicon nanoparticles reduce glioblastoma cell migration across tight extracellular space. Sci Rep. 2020;10(1):2320. 32047170 10.1038/s41598-020-59146-5 PMC7012928

[63] Guilhamon P, Chesnelong C, Kushida MM, Nikolic A, Singhal D, MacLeod G, et al. Single-cell chromatin accessibility profiling of glioblastoma identifies an invasive cancer stem cell population associated with lower survival. Elife. 2021;10:e64090. 33427645 10.7554/eLife.64090 PMC7847307

[64] Koh L, Novera W, Lim SW, Chong YK, Pang QY, Low D, et al. Integrative multi-omics approach to targeted therapy for glioblastoma. Pharmacol Res. 2022;182:106318. 35714825 10.1016/j.phrs.2022.106308

[65] Cachia D, Kamiya-Matsuoka C, Mandel JJ, Olar A, Cykowski MD, Armstrong TS, et al. Primary and secondary gliosarcomas: clinical, molecular and survival characteristics. J Neurooncol. 2015;125(2):401–10. 26354773 10.1007/s11060-015-1930-y PMC8323586

[66] Hegi ME, Diserens AC, Gorlia T, Hamou MF, de Tribolet N, Weller M, et al. MGMT gene silencing and benefit from temozolomide in glioblastoma. N Engl J Med. 2005;352(10):997–1003. 15758010 10.1056/NEJMoa043331

[67] Prasad G, Sottero T, Yang X, Mueller S, James CD, Weiss WA, et al. Inhibition of PI3K/mTOR pathways in glioblastoma and implications for combination therapy with temozolomide. Neuro Oncol. 2011;13(4):384–92. 21317208 10.1093/neuonc/noq193 PMC3064692

[68] Lam FC, Morton SW, Wyckoff J, Vu Han TL, Hwang MK, Maffa A, et al. Enhanced efficacy of combined temozolomide and bromodomain inhibitor therapy for gliomas using targeted nanoparticles. Nat Commun. 2018;9(1):1991. 29777137 10.1038/s41467-018-04315-4 PMC5959860

[69] Nitta Y, Shimizu S, Shishido-Hara Y, Suzuki K, Shiokawa Y, Nagane M. Nimotuzumab enhances temozolomide-induced growth suppression of glioma cells expressing mutant EGFR in vivo. Cancer Med. 2016;5(3):486–99. 26778701 10.1002/cam4.614 PMC4799951

[70] Liu YJ, Ma YC, Zhang WJ, Yang ZZ, Liang DS, Wu ZF, et al. Combination therapy with micellarized cyclopamine and temozolomide attenuates glioblastoma growth through Gli1 down-regulation. Oncotarget. 2017;8(26):42495–509. 28477008 10.18632/oncotarget.17205 PMC5522083

[71] Wang R, Chadalavada K, Wilshire J, Kowalik U, Hovinga KE, Geber A, et al. Glioblastoma stem-like cells give rise to tumour endothelium. Nature. 2010;468(7325):829–33. 21102433 10.1038/nature09624

[72] Iorio AL, da Ros M, Genitori L, Lucchesi M, Colelli F, Signorino G, et al. Tumor response of temozolomide in combination with morphine in a xenograft model of human glioblastoma. Oncotarget. 2017;8(52):89595–606. 29163773 10.18632/oncotarget.19875 PMC5685694

[73] Zeng J, See AP, Phallen J, Jackson CM, Belcaid Z, Ruzevick J, et al. Anti-PD-1 blockade and stereotactic radiation produce long-term survival in mice with intracranial gliomas. Int J Radiat Oncol Biol Phys. 2013;86(2):343–9. 23462419 10.1016/j.ijrobp.2012.12.025 PMC3963403

[74] Raychaudhuri B, Rayman P, Huang P, Grabowski M, Hambardzumyan D, Finke JH, et al. Myeloid derived suppressor cell infiltration of murine and human gliomas is associated with reduction of tumor infiltrating lymphocytes. J Neurooncol. 2015;122(2):293–301. 25579983 10.1007/s11060-015-1720-6

[75] Parsons DW, Jones S, Zhang X, Lin JCH, Leary RJ, Angenendt P, et al. An integrated genomic analysis of human glioblastoma multiforme. Science. 2008;321(5897):1807–12. 18772396 10.1126/science.1164382 PMC2820389

[76] Guo X, Gu L, Li Y, Zheng Z, Chen W, Wang Y, et al. Histological and molecular glioblastoma, IDH-wildtype: a real-world landscape using the 2021 WHO classification of central nervous system tumors. Front Oncol. 2023;13:1213149. 10.3389/fonc.2023.1200815 PMC10358772 37483487

[77] Butler M, Pongor L, Su YT, Xi L, Raffeld M, Quezado M, et al. MGMT status as a clinical biomarker in glioblastoma. Trends Cancer. 2020;6(5):380–91. 32348734 10.1016/j.trecan.2020.02.010 PMC7315323

[78] Zhang Y, Dube C, Gibert M, Cruickshanks N, Wang B, Coughlan M, et al. The p53 pathway in glioblastoma. Cancers. 2018;10(9):297. 30200436 10.3390/cancers10090297 PMC6162501

[79] Tang F, Chen X, Liu JS, Liu ZY, Yang JZ, Wang ZF, et al. TERT mutations-associated alterations in clinical characteristics, immune environment, and therapy response in glioblastomas. Discover Oncol. 2023;14(1):148. 10.1007/s12672-023-00760-w PMC10421840 37566174

[80] Shboul SA, Boyle S, Singh A, Saleh T, AlRjoub M, Al Karsaneh OA, et al. FISH-based investigation of CDKN2A and IFNA14 in glioblastoma. Neuro Oncol. 2023;25(Suppl 3):18.

[81] Chang M, Sherief M, Ioannou M, Chinnasamy V, Chen L, Frost M, et al. NF1 expression profiling in IDH-wildtype glioblastoma: genomic associations and survival outcomes. Acta Neuropathol Commun. 2024;12(1):172. 39472976 10.1186/s40478-024-01875-z PMC11520828

[82] Hagemann C, Anacker J, Haas S, Riesner D, Schömig B, Ernestus RI, et al. Comparative expression pattern of matrix-metalloproteinases in human glioblastoma cell lines and primary cultures. BMC Res Notes. 2010;3(1):293. 21067565 10.1186/1756-0500-3-293 PMC2996401

[83] Olmez OF, Cubukcu E, Evrensel T, Kurt M, Avci N, Tolunay S, et al. The immunohistochemical expression of c-Met is an independent predictor of survival in patients with glioblastoma multiforme. Clin Transl Oncol. 2014;16(2):173–7. 23740136 10.1007/s12094-013-1059-4

[84] Wang H, Mao X, Ye L, Cheng H, Dai X. The role of the S100 protein family in glioma. J Cancer. 2022;13(10):3022–30. 36046652 10.7150/jca.73365 PMC9414020

[85] Seyhan AA. Circulating liquid biopsy biomarkers in glioblastoma: advances and challenges. Int J Mol Sci. 2024;25(14):7974. 39063215 10.3390/ijms25147974 PMC11277426

[86] Wang X, Wang L, Lin H, Zhu Y, Huang D, Lai M, et al. Research progress of CTC, ctDNA, and EVs in cancer liquid biopsy. Front Oncol. 2024;14:1303335. 38333685 10.3389/fonc.2024.1303335 PMC10850354

[87] Gatto L, Franceschi E, Di Nunno V, Tosoni A, Lodi R, Brandes AA. Liquid biopsy in glioblastoma management: from current research to future perspectives. Oncologist. 2021;26(10):865–78. 34105205 10.1002/onco.13858 PMC8488799

[88] Müller Bark J, Kulasinghe A, Chua B, Day BW, Punyadeera C. Circulating biomarkers in patients with glioblastoma. Br J Cancer. 2020;122(3):295–305. 31666668 10.1038/s41416-019-0603-6 PMC7000822

[89] Bryukhovetskiy I. Cell-based immunotherapy of glioblastoma multiforme. Oncol Lett. 2022;23(4):1–14. 35251352 10.3892/ol.2022.13253 PMC8895466

[90] Johnson KC, Anderson KJ, Courtois ET, Gujar AD, Barthel FP, Varn FS, et al. Single-cell multimodal glioma analyses identify epigenetic regulators of cellular plasticity and environmental stress response. Nat Genet. 2021;53(10):1456–68. 34594038 10.1038/s41588-021-00926-8 PMC8570135

[91] Neftel C, Laffy J, Filbin MG, Hara T, Shore ME, Rahme GJ, et al. An integrative model of cellular states, plasticity, and genetics for glioblastoma. Cell. 2019;178(4):835-849.e21. 31327527 10.1016/j.cell.2019.06.024 PMC6703186

[92] Ravi VM, Will P, Kueckelhaus J, Sun N, Joseph K, Salié H, et al. Spatially resolved multi-omics deciphers bidirectional tumor-host interdependence in glioblastoma. Cancer Cell. 2022;40(6):639-655.e13. 35700707 10.1016/j.ccell.2022.05.009

[93] Wang L, Jung J, Babikir H, Shamardani K, Jain S, Feng X, et al. A single-cell atlas of glioblastoma evolution under therapy reveals cell-intrinsic and cell-extrinsic therapeutic targets. Nat Cancer. 2022;3(12):1534–52. 36539501 10.1038/s43018-022-00475-x PMC9767870

[94] Deacon S, Cahyani I, Holmes N, Fox G, Munro R, Wibowo S, Murray T, Mason H, Housley M, Martin D, Sharif A. ROBIN: a unified nanopore-based sequencing assay integrating real-time, intraoperative methylome classification and next-day comprehensive molecular brain tumour profiling for ultra-rapid tumour diagnostics. medRxiv. 2024;7:2024–109. 10.1093/neuonc/noaf103 PMC12448888 40392954

[95] Chen PC, Gadepalli K, MacDonald R, Liu Y, Kadowaki S, Nagpal K, et al. An augmented reality microscope with real-time artificial intelligence integration for cancer diagnosis. Nat Med. 2019;25(9):1453–7. 10.1038/s41591-019-0539-7. 31406351 10.1038/s41591-019-0539-7

[96] Wishart DS, Knox C, Guo AC, Shrivastava S, Hassanali M, Stothard P, et al. DrugBank: a comprehensive resource for in silico drug discovery and exploration. Nucleic Acids Res. 2006;34:D668–72. 10.1093/nar/gkj067. 16381955 10.1093/nar/gkj067 PMC1347430

[97] Zhang L, Tan J, Han D, Zhu H. From machine learning to deep learning: progress in machine intelligence for rational drug discovery. Drug Discov Today. 2017;22(11):1680–5. 28881183 10.1016/j.drudis.2017.08.010

[98] Young JD, Cai C, Lu X. Unsupervised deep learning reveals prognostically relevant subtypes of glioblastoma. BMC Bioinform. 2017;18(Suppl 11):381. 10.1186/s12859-017-1798-2 PMC5629551 28984190

[99] Ricciotti E, FitzGerald GA. Aspirin in the prevention of cardiovascular disease and cancer. Annu Rev Med. 2021;72:473–95. 33502899 10.1146/annurev-med-051019-102940

[100] Singhal S, Mehta J, Desikan R, Ayers D, Roberson P, Eddlemon P, et al. Antitumor activity of thalidomide in refractory multiple myeloma. N Engl J Med. 1999;341(21):1565–71. 10564685 10.1056/NEJM199911183412102

[101] Weth FR, Hoggarth GB, Weth AF, Paterson E, White MPJ, Tan ST, et al. Unlocking hidden potential: advancements, approaches, and obstacles in repurposing drugs for cancer therapy. Br J Cancer. 2024;130:703–15. 38012383 10.1038/s41416-023-02502-9 PMC10912636

[102] Liu KH, Yang ST, Lin YK, Lin JW, Lee YH, Wang JY, et al. Fluoxetine, an antidepressant, suppresses glioblastoma by evoking AMPAR-mediated calcium-dependent apoptosis. Oncotarget. 2015;6(7):5088–100. 25671301 10.18632/oncotarget.3243 PMC4467135

[103] Guryanova OA, Wu Q, Cheng L, Lathia JD, Huang Z, Yang J, et al. Nonreceptor tyrosine kinase BMX maintains self-renewal and tumorigenic potential of glioblastoma stem cells by activating STAT3. Cancer Cell. 2011;19(4):498–511. 21481791 10.1016/j.ccr.2011.03.004 PMC3076106

[104] Chen Y, Zhao S, Fan Z, Li Z, Zhu Y, Shen T, et al. Metformin attenuates plaque-associated tau pathology and reduces amyloid-β burden in APP/PS1 mice. Alzheimers Res Ther. 2021;13(1):40. 33563332 10.1186/s13195-020-00761-9 PMC7871393

[105] Kumar A, Prakash A, Pahwa D, Mishra J. Montelukast potentiates the protective effect of rofecoxib against kainic acid-induced cognitive dysfunction in rats. Pharmacol Biochem Behav. 2012;103(1):43–52. 22878042 10.1016/j.pbb.2012.07.015

[106] Bayram E, Marras C, Standaert DG, Kluger BM, Bordelon YM, Shprecher DR, et al. Progressive supranuclear palsy and statin use. Mov Disord. 2020;35(7):1253–7. 32220093 10.1002/mds.28038 PMC7375943

[107] Frank S, Testa CM, Stamler D, Kayson E, Davis C, Edmondson MC, et al. Effect of deutetrabenazine on chorea among patients with Huntington disease. JAMA. 2016;316(1):40. 27380342 10.1001/jama.2016.8655

[108] Jankovic J, Clarence-Smith K. Tetrabenazine for the treatment of chorea and other hyperkinetic movement disorders. Expert Rev Neurother. 2011;11(11):1509–23. 22014129 10.1586/ern.11.149

[109] Sandouka S, Singh PK, Saadi A, Taiwo RO, Sheeni Y, Zhang T, et al. Repurposing dimethyl fumarate as an antiepileptogenic and disease-modifying treatment for drug-resistant epilepsy. J Transl Med. 2023;21(1):796. 37940957 10.1186/s12967-023-04695-2 PMC10634153

[110] El-Azab MF, Moustafa YM. Influence of calcium channel blockers on anticonvulsant and antinociceptive activities of valproic acid in pentylenetetrazole-kindled mice. Pharmacol Rep. 2012;64(2):305–14. 22661180 10.1016/s1734-1140(12)70769-7

[111] Nizzardo M, Nardini M, Ronchi D, Salani S, Donadoni C, Fortunato F, et al. Beta-lactam antibiotic offers neuroprotection in a spinal muscular atrophy model by multiple mechanisms. Exp Neurol. 2011;229(2):214–25. 21295027 10.1016/j.expneurol.2011.01.017

[112] Bartek J, Hodny Z. Dopamine signaling: target in glioblastoma. Oncotarget. 2014;5(5):1116–7. 24657925 10.18632/oncotarget.1835 PMC4012725

[113] Kang S, Hong J, Lee JM, Moon HE, Jeon B, Choi J, et al. Trifluoperazine, a well-known antipsychotic, inhibits glioblastoma invasion by binding to calmodulin and disinhibiting calcium release channel IP3R. Mol Cancer Ther. 2017;16(1):217–27. 28062709 10.1158/1535-7163.MCT-16-0169-T

[114] Karbownik MS, Szemraj J, Wieteska Ł, Antczak A, Górski P, Kowalczyk E, et al. Antipsychotic drugs differentially affect mRNA expression of genes encoding the neuregulin 1-downstream ErbB4-PI3K pathway. Pharmacology. 2016;98(1–2):4–12. 26960157 10.1159/000444534

[115] Karpel-Massler G, Kast RE, Westhoff MA, Dwucet A, Welscher N, Nonnenmacher L, et al. Olanzapine inhibits proliferation, migration and anchorage-independent growth in human glioblastoma cell lines and enhances temozolomide’s antiproliferative effect. J Neurooncol. 2015;122(1):21–33. 25524815 10.1007/s11060-014-1688-7

[116] Belagaje SR, Brahmbhatt D, Abramoff BA. Selective serotonin reuptake inhibitors. In: Ischemic stroke therapeutics. Cham: Springer International Publishing; 2024. p. 313–21.

[117] Higgins SC, Pilkington GJ. The in vitro effects of tricyclic drugs and dexamethasone on cellular respiration of malignant glioma. Anticancer Res. 2010;30(2):391–7. 20332444

[118] Bielecka-Wajdman AM, Lesiak M, Ludyga T, Sieroń A, Obuchowicz E. Reversing glioma malignancy: a new look at the role of antidepressant drugs as adjuvant therapy for glioblastoma multiforme. Cancer Chemother Pharmacol. 2017;79(6):1249–56. 28500556 10.1007/s00280-017-3329-2

[119] Hayashi K, Michiue H, Yamada H, Takata K, Nakayama H, Wei FY, et al. Fluvoxamine, an antidepressant, inhibits human glioblastoma invasion by disrupting actin polymerization. Sci Rep. 2016;6(1):23372. 26988603 10.1038/srep23372 PMC4796892

[120] Tzadok S, Beery E, Israeli M, Uziel O, Lahav M, Fenig E, Gil-Ad I, Weizman A, Nordenberg J. In vitro novel combinations of psychotropics and anti-cancer modalities in U87 human glioblastoma cells. Int J Oncol. 2010;37(4):1043–51. 20811727 10.3892/ijo_00000756

[121] Van Nifterik KA, Van den Berg J, Slotman BJ, Lafleur MVM, Sminia P, Stalpers LJA. Valproic acid sensitizes human glioma cells for temozolomide and γ-radiation. J Neurooncol. 2012;107(1):61–7. 22037799 10.1007/s11060-011-0725-z

[122] Lee CY, Lai HY, Chiu A, Chan SH, Hsiao LP, Lee ST. The effects of antiepileptic drugs on the growth of glioblastoma cell lines. J Neurooncol. 2016;127(3):445–53. 26758059 10.1007/s11060-016-2056-6 PMC4835521

[123] Pinheiro R, Braga C, Santos G, Bronze MR, Perry MJ, Moreira R, et al. Targeting gliomas: can a new alkylating hybrid compound make a difference? ACS Chem Neurosci. 2017;8(1):50–9. 27665765 10.1021/acschemneuro.6b00169

[124] Triscott J, Rose Pambid M, Dunn SE. Concise review: bullseye: targeting cancer stem cells to improve the treatment of gliomas by repurposing disulfiram. Stem Cells. 2015;33(4):1042–6. 25588723 10.1002/stem.1956

[125] Triscott J, Lee C, Hu K, Fotovati A, Berns R, Pambid M, et al. Disulfiram, a drug widely used to control alcoholism, suppresses self-renewal of glioblastoma and overrides resistance to temozolomide. Oncotarget. 2012;3(10):1112–23. 23047041 10.18632/oncotarget.604 PMC3717961

[126] Krott Z, Mistrik M, Andersen KK, Friis S, Majera D, Gursky J, et al. Alcohol-abuse drug disulfiram targets cancer via p97 segregase adaptor NPL4. Nature. 2017;552(7684):194–9. 29211715 10.1038/nature25016 PMC5730499

[127] De Witt M, Gamble A, Hanson D, Markowitz D, Powell C, Al Dimassi S, et al. Repurposing mebendazole as a replacement for vincristine for the treatment of brain tumors. Mol Med. 2017;23(1):50–6. 28386621 10.2119/molmed.2017.00011 PMC5403762

[128] Zheng M, Sun W, Gao S, Luan S, Li D, Chen R, et al. Structure-based discovery of clomifene as a potent inhibitor of cancer-associated mutant IDH1. Oncotarget. 2017;8(27):44255–65. 28498812 10.18632/oncotarget.17464 PMC5546478

[129] Molenaar RJ, Coelen RJ, Khurshed M, Roos E, Caan MW, van Linde ME, et al. Study protocol of a phase IB/II clinical trial of metformin and chloroquine in patients with IDH1-mutated or IDH2-mutated solid tumours. BMJ Open. 2017;7(6):e014961. 28601826 10.1136/bmjopen-2016-014961 PMC5541450

[130] Ferla R, Haspinger E, Surmacz E. Metformin inhibits leptin-induced growth and migration of glioblastoma cells. Oncol Lett. 2012;4(5):1077–81. 23162655 10.3892/ol.2012.843 PMC3499461

[131] Kast R, Karpel-Massler G, Halatsch ME. Can the therapeutic effects of temozolomide be potentiated by stimulating AMP-activated protein kinase with olanzapine and metformin? Br J Pharmacol. 2011;164(5):1393–6. 21410456 10.1111/j.1476-5381.2011.01320.x PMC3221093

[132] Sesen J, Dahan P, Scotland SJ, Saland E, Dang VT, Lemarié A, et al. Metformin inhibits growth of human glioblastoma cells and enhances therapeutic response. PLoS ONE. 2015;10(4):e0123721. 25867026 10.1371/journal.pone.0123721 PMC4395104

[133] Lee YS, Dutta A. The tumor suppressor microRNA let-7 represses the HMGA2 oncogene. Genes Dev. 2007;21(9):1025–30. 17437991 10.1101/gad.1540407 PMC1855228

[134] Gritti M, Würth R, Angelini M, Barbieri F, Peretti M, Pizzi E, et al. Metformin repositioning as antitumoral agent: Selective antiproliferative effects in human glioblastoma stem cells, via inhibition of CLIC1-mediated ion current. Oncotarget. 2014;5(22):11252–68. 25361004 10.18632/oncotarget.2617 PMC4294381

[135] Gaist D, Hallas J, Friis S, Hansen S, Sørensen HT. Statin use and survival following glioblastoma multiforme. Cancer Epidemiol. 2014;38(6):722–7. 25455652 10.1016/j.canep.2014.09.010

[136] Yanae M, Tsubaki M, Satou T, Itoh T, Imano M, Yamazoe Y, et al. Statin-induced apoptosis via the suppression of ERK1/2 and Akt activation by inhibition of the geranylgeranyl-pyrophosphate biosynthesis in glioblastoma. J Exp Clin Cancer Res. 2011;30(1):74. 21831290 10.1186/1756-9966-30-74 PMC3163617

